# A volumetric three-dimensional digital light photoactivatable dye display

**DOI:** 10.1038/ncomms15239

**Published:** 2017-07-11

**Authors:** Shreya K. Patel, Jian Cao, Alexander R. Lippert

**Affiliations:** 1 Department of Chemistry, Southern Methodist University, 3215 Daniel Avenue, Dallas, Texas 75275-0314, USA; 2 Center for Drug Discovery, Design, and Delivery (CD4), Southern Methodist University, 3215 Daniel Avenue, Dallas, Texas 75275-0314, USA; 3 Center for Global Health Impact (CGHI), Southern Methodist University, 3215 Daniel Avenue, Dallas, Texas 75275-0314, USA

## Abstract

Volumetric three-dimensional displays offer spatially accurate representations of images with a 360° view, but have been difficult to implement due to complex fabrication requirements. Herein, a chemically enabled volumetric 3D digital light photoactivatable dye display (3D Light PAD) is reported. The operating principle relies on photoactivatable dyes that become reversibly fluorescent upon illumination with ultraviolet light. Proper tuning of kinetics and emission wavelengths enables the generation of a spatial pattern of fluorescent emission at the intersection of two structured light beams. A first-generation 3D Light PAD was fabricated using the photoactivatable dye *N*-phenyl spirolactam rhodamine B, a commercial picoprojector, an ultraviolet projector and a custom quartz imaging chamber. The system displays a minimum voxel size of 0.68 mm^3^, 200 μm resolution and good stability over repeated ‘on-off’ cycles. A range of high-resolution 3D images and animations can be projected, setting the foundation for widely accessible volumetric 3D displays.

## Abstract (web summary)

Despite living in a three-dimensional world, almost all information in our society is conveyed in a two-dimensional format. Here, the authors provide a technique for the generation of spatially accurate and high-resolution three-dimensional images using fluorescent photoswitch chemistry.

Advanced imaging techniques can now acquire high-resolution biological imaging data in three dimensions^1^,^2^,^3^, but digital methods to display this data are largely restricted to two dimensions. Three-dimensional (3D) stereoscopic movies are ubiquitous in the entertainment industry, but find limited use in other sectors because they are actually two-dimensional (2D) images that rely on binocular disparity to trick the brain into seeing a 3D image^4^. Volumetric 3D displays are spatially accurate and truly 3D platforms that have expanded utility for medical imaging, engineering and architecture, education, and military applications^5^. The classical 2D representation of 3D images was originally optimized by renaissance artists/mathematicians^6^ and relies on psychological depth cues, including linear perspective, occlusion, shading and texture. By contrast, volumetric 3D displays truly structure light in a 3D space and fully integrate physical depth cues, including accommodation (differences in eye muscle tension when focusing at different distances), convergence (angle of the eyes’ focus at different distances), motion parallax (closer objects appear to move faster) and binocular disparity (differences in the images seen by the left eye and right eye)^4^. Volumetric 3D displays can be characterized into swept-volume displays that include high-speed image projection onto moving screens^7^,^8^,^9^ and rotating light-emitting diodes (LEDs)^10^, and static volume displays that include active matrix display units^11^ and beam-addressable static volume displays^12^,^13^,^14^. Beam-addressable static volume displays typically use beams of high-powered light to induce a specialized static volume to emit light at spatially defined points in 3D space called voxels. These systems are attractive from a manufacturing viewpoint due to the lack of moving parts and drastic simplification in the arrangement of voxels. Beam-addressable systems can employ upconversion processes^10^,^11^ or plasma generation^12^, but these techniques require expensive high-energy beams that pose non-trivial safety risks. Photochromic holography^15^,^16^,^17^,^18^,^19^ has been explored and a photochromic 3D display was disclosed by the Battelle Development Corporation in the late 1960s (refs 20, 21), but a successful system was never commercialized to the best of our knowledge. The need for complex fabrication, moving parts, high-speed projectors or high laser powers has posed a significant obstacle to widespread dissemination of volumetric 3D display technology.

Herein, we report a 3D Light PAD that uses a special class of photoactivatable molecules and digital light processing (DLP) technology to generate structured light in three dimensions. Photoactivatable molecules^22^,^23^,^24^,^25^,^26^ are central components of groundbreaking advances in super-resolution microscopy^27^,^28^,^29^,^30^,^31^, data storage^32^,^33^, molecular motors^34^,^35^,^36^,^37^ and photopharmacology^38^,^39^,^40^,^41^,^42^. Several classes of photoactivatable molecules have been described, including diarylethenes^43^,^44^, diarylazo compounds^33^,^45^, spiropyran/merocyanines^46^, oxazines^2^,^26^, photocaged compounds^25^,^35^,^37^,^47^,^48^,^49^, BF_2_-azo complexes^50^,^51^ and spirolactam rhodamines^23^,^24^,^52^,^53^,^54^. These molecules undergo a photochemical reaction to toggle between at least two different molecular states that can vary in shape, colour, fluorescence and other molecular properties. If this reaction is reversible, then these compounds are referred to as photoswitches. Molecular scaffolds and chemical systems have been developed and optimized in efforts to tune the rates of photoactivating reactions^55^, reversibility kinetics^56^, absorption/emission wavelengths^40^ and repeatability cycles^57^.

The operating principle of a 3D Light PAD rests on using small photoactivatable molecules with a particular set of optical properties: a non-fluorescent ‘off’ state, a fluorescent or visible absorbing ‘on’ state that can be accessed by illuminating at a wavelength outside of the visible absorbance spectrum, a fast rate of photoactivation and a fast deactivation to the ‘off’ state. In photoactivated localization microscopy^58^,^59^ and 3D stochastic optical reconstruction microscopy^60^, small subsets of fluorophores are sequentially activated and located by point spread function analysis to construct a 3D data set from a large series of images. In contrast, a 3D Light PAD simultaneously activates and excites fluorophores in a spatial pattern defined by DLP to generate a viewable and spatially accurate volumetric 3D image. *N*-substituted spirolactam rhodamines are a class of photoactivatable compounds that can be switched from a non-fluorescent to a fluorescent isomer using ultraviolet light^23^,^24^,^47^,^48^,^49^. This photochemical reaction is thermally reversible, and the fluorescent isomers have absorption and emission in the visible region of the spectrum^47^,^49^. We envisioned that under the appropriate conditions a 3D voxel could be generated by illumination of these photoactivatable dyes dispersed in a low-scattering solvent or matrix with two different wavelengths of light.

## Results

### Characterization of *N*-phenyl spirolactam rhodamine B

**Figure 1:**
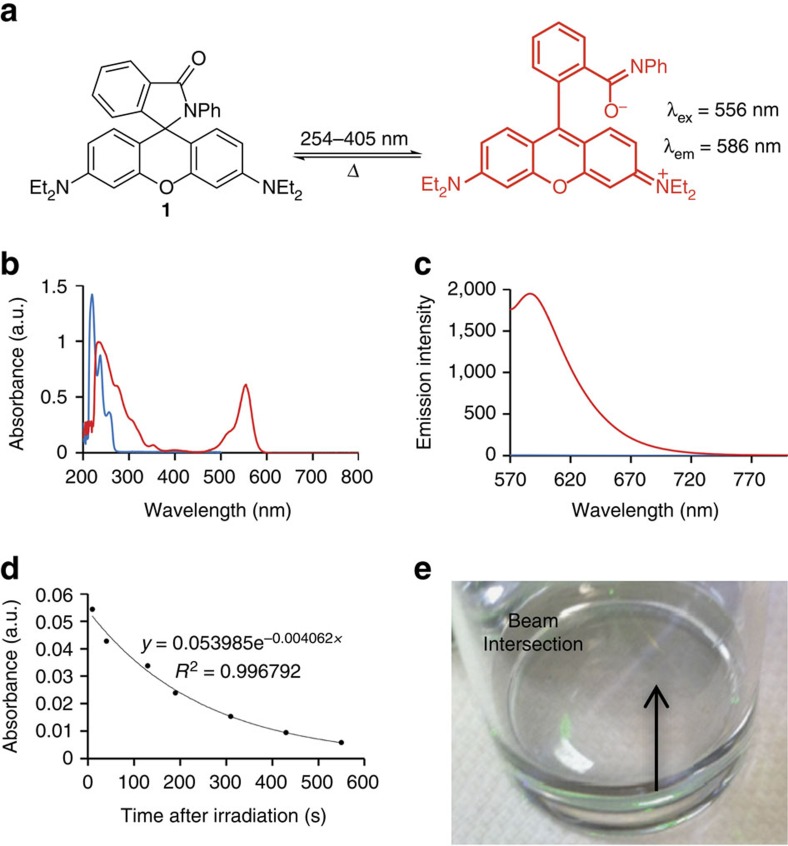
Spectroscopic properties of *N*-phenyl spirolactam rhodamine B **1**. (**a**) Reaction scheme for photoactivatable fluorescence of **1**. (**b**) Absorbance spectrum of 50 μM **1** in CH_2_Cl_2_ before (blue trace) and after (red trace) irradiating with 0.26 mW cm^−2^ 254 nm light for 5 min. (**c**) Emission spectrum of 50 μM **1** in CH_2_Cl_2_ before (blue trace) and after (red trace) irradiating with 0.26 mW cm^−2^ 254 nm light for 5 min. *λ*_ex_=556 nm. (**d**) Thermal fading kinetics of the absorption at 556 nm of 5 mM **1** in CH_2_Cl_2_ after irradiating with 1.0 mW cm^−2^ 315 nm light for 90 s. (**e**) Image of a vial containing 5 mM **1** in CH_2_Cl_2_ and two intersecting beams from 532 nm and a 405 nm laser pointers. Absorbance data is reported in arbitrary units (a.u.).

*N*-phenyl spirolactam rhodamine B **1** was synthesized by POCl_3_-mediated formation of the acid chloride followed by acyl substitution with aniline (Fig. 1a, Supplementary Figs 1–3). Purification by silica column chromatography and recrystallization from 3:1 hexane:ethyl acetate provided pure white crystals needed for low background fluorescence and optimal performance in a 3D Light PAD device. Before illumination, the absorbance spectrum of **1** shows peaks at 240, 274 and 314 nm, with no observable absorbance above 400 nm (Fig. 1b, blue trace), and very weak background fluorescence when excited at 556 nm (Fig. 1c, blue trace). In contrast, a strong peak in the absorbance spectrum appears at 556 nm after illumination of **1** in a quartz cuvette for 5 min with 254 nm light, as well as other peaks at 240, 274, 308, 353 and 404 nm (Fig. 1b, red trace). This increase in absorbance shows a linear dependence on illumination time (Supplementary Fig. 4) and is greater when using higher frequency illumination (Supplementary Fig. 5). Exciting at 556 nm with the dye in its ‘on’ state provided a strong emission centred at 586 nm (Fig. 1c, red trace). Acquiring emission spectra of the ‘on’ and ‘off’ states of **1** under identical settings revealed ∼415-fold increase in emission intensity for the post-ultraviolet-illuminated samples. The rate of the thermal fading reaction was examined by monitoring the decrease in the absorbance at 556 nm after cessation of illumination (Fig. 1d). The decay was fit to a single exponential and a *k*_obs_=0.0041, s^–1^ was determined. Having spectroscopically confirmed the photoswitching properties, a preliminary demonstration of voxel generation was performed using a 5 mM solution of **1** in dichloromethane in a vial (Fig. 1e). A 532 nm laser pointer (5 mW) and a 405 nm laser pointer (5 mW) were aimed to intersect in the vial, revealing an illuminated point of light with increased brightness at the intersection. This preliminary demonstration motivated work towards the optimization and prototyping of a first-generation 3D Light PAD device.

### Triethylamine increases the rate of thermal fading

**Figure 2:**
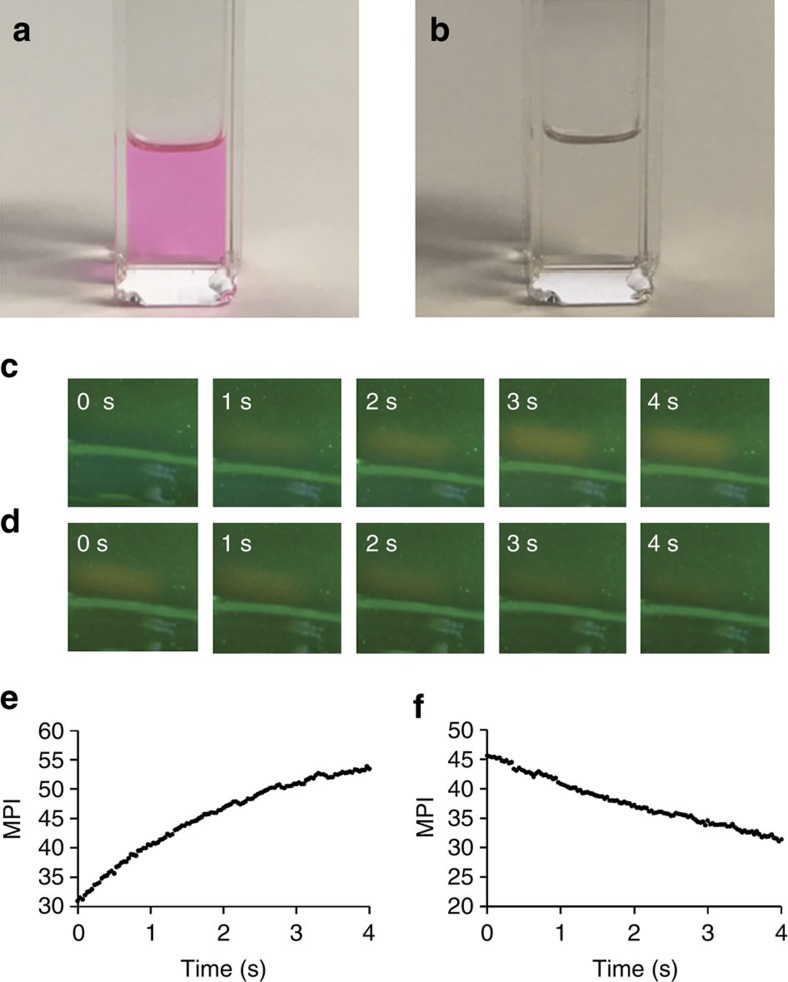
Substoichiometric triethylamine increases the rate of thermal fading. (**a**) Image of 5 mM **1** in CH_2_Cl_2_ after irradiating with 0.26 mW cm^−2^ 254 nm light for 5 min. (**b**) Image of 5 mM **1** in CH_2_Cl_2_ after irradiating with 0.26 mW cm^−2^ 254 nm light for 5 min, followed by addition of 1 drop of triethylamine. (**c**) Images of 5 mM **1** in CH_2_Cl_2_ and 5 p.p.m. triethylamine (36 μM) irradiated with 0.40 mW cm^−2^ 385 nm and 2.0 mW cm^−2^ 525 nm light for 0, 1, 2, 3 and 4 s. (**d**) Images of 5 mM **1** in CH_2_Cl_2_ and 5 p.p.m. triethylamine (36 μM) after irradiating with 0.40 mW cm^−2^ 385 nm and 2.0 mW cm^−2^ 525 nm light for 4 s, and then removing the 385 nm irradiation for 0, 1, 2, 3 and 4 s. (**e**) Plot of MPI of illuminated cross section versus time for the photoactivation experiment shown in **c**. (**f**) Plot of MPI of irradiated cross section versus time for the thermal fading experiment shown in **d**.

Expanding from the demonstration of preliminary voxel formation to full 3D image generation was initially plagued by slow thermal fading kinetics. It was observed that increased ultraviolet illumination time resulted in slower thermal fading (Supplementary Fig. 6), and illuminated solutions of **1** in CHCl_3_ or CDCl_3_ would irreversibly become coloured and fluorescent. We hypothesized that addition of a base may increase the reversibility of photoactivation in light of literature precedent for acid-mediated ring opening of *N*-aryl spirolactam rhodamines and other structures related to **1** (refs 61, 62). Adding a drop of triethylamine to a photoactivated solution of **1** that was resistant to thermal fading resulted instantaneously in a clear solution (Fig. 2a,b). At this stage, we proceeded to investigate this effect in more detail and turned towards DLP projector systems as light sources as opposed to the laser pointers used in Fig. 1e. A commercial Miroir 720p DLP picoprojector was used as a visible light source, and a Pro4500 DLP projector equipped with a 385 nm LED was used for the ultraviolet light source. The projectors were aligned so the green and ultraviolet beams would cross in the solution of **1** in a vial. In this way, the timing could be efficiently controlled, and the illumination could be recorded using a standard CMOS camera. Solutions of 5 mM **1** in dichloromethane were prepared containing 1 p.p.m. (7.2 μM), 5 p.p.m. (36 μM) and 15 p.p.m. (108 μM) triethylamine. The vial was illuminated with the green and ultraviolet projectors, and a time course of the turn-on was acquired (Fig. 2c and Supplementary Figs 7 and 8). Switching off the ultraviolet beam then allowed real-time monitoring of the thermal fading reaction (Fig. 2d and Supplementary Figs 7 and 8). A video was recorded at 30 frames per second and analysis of the mean pixel intensity (MPI) in ImageJ enabled kinetic traces with ∼33 ms resolution (Fig. 2e,f and Supplementary Figs 7 and 8). The off-rate was fit to a single exponential and revealed drastically increased rates with *k*_obs_=0.048, 0.20 and 0.50 s^–1^ for 1, 5 and 15 p.p.m. triethylamine, respectively. The increase in rate was linear with respect to the triethylamine concentration (Supplementary Fig. 9) and resulted in reduced contrast at higher triethylamine concentrations (Supplementary Fig. 10). We confirmed that this effect is not due to covalent adduct formation with the xanthene core, as the sterically hindered base di-isopropylethylamine also mediated thermal fading (Supplementary Fig. 11) and no clear adduct is observable by ^1^H NMR spectroscopy, even with the addition of excess triethylamine to an irradiated sample (Supplementary Fig. 12). Additionally, we investigated the possibility of collisional quenching of triethylamine by monitoring the decrease in fluorescence emission of photoactivated **1**, rhodamine B and rosamine (Supplementary Fig. 13). While rhodamine B can participate in a similar isomerization reaction as photoactivated **1**, rosamine lacks the carboxylic acid and cannot undergo isomerization. The fluorescence emission of both photoactivated **1** and rhodamine B was almost completely eliminated with 100 μM triethylamine (Supplementary Fig. 13a,b). On the other hand, rosamine displayed only minimal quenching even with as high as 1 mM triethylamine (Supplementary Fig. 13c). Taken together, these experiments suggest that the effect of triethylamine on thermal fading is due to promotion of the isomerization reaction and not due to adduct formation or collisional quenching. These key results provided a method to tune the thermal fading kinetics, ultimately enabling image generation.

### Imaging system design and evaluation of optical filters

**Figure 3:**
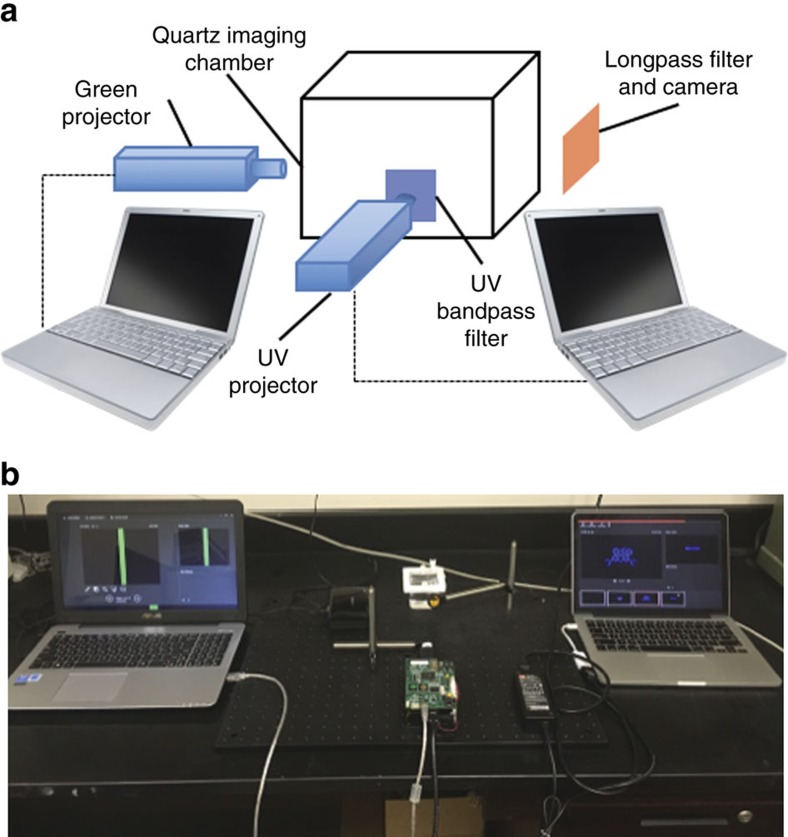
Design of a first-generation 3D Light PAD. (**a**) Schematic of the system architecture. (**b**) Photograph of the first-generation 3D Light PAD.

**Figure 4:**
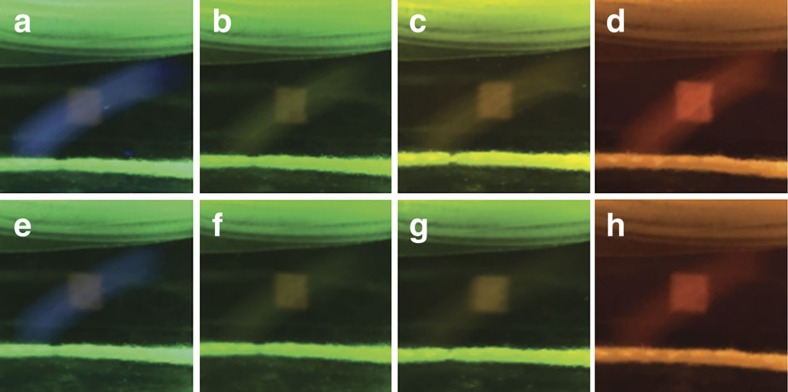
Optical filters improve image quality. A ultraviolet square was projected from the Pro4500 ultraviolet projector and a green bar was projected from the Miroir 720p picoprojector into a solution of **1** in CH_2_Cl_2_ containing 1 p.p.m. (7.2 μM) triethylamine using (**a**) no filters, (**b**) 515 nm longpass filter, (**c**) 550 nm longpass filter, (**d**) 590 nm longpass filter, (**e**) ultraviolet bandpass filter (240–395 nm), (**f**) ultraviolet bandpass filter (240–395 nm) and 515 nm longpass filter, (**g**) ultraviolet bandpass filter (240–395 nm) and 550 nm longpass filter, (**h**) ultraviolet bandpass filter (240–395 nm) and 590 nm longpass filter. Images were generated using 2.0 mW cm^−2^ 525 nm light and 0.40 mW cm^−2^ 385 nm light (no bandpass filter) or 0.27 mW cm^−2^ 385 nm light (with bandpass filter).

With the ability to tune the thermal fading kinetics, a first-generation 3D Light PAD was designed for 3D image generation (Fig. 3). The graphics engine consists of two personal computers to control a Miroir 720p picoprojector, and a Pro4500 projector equipped with a 385 nm ultraviolet LED and a 525 nm green LED. Initial imaging experiments were performed in a small glass jar or vial, but higher-quality images were acquired using a custom-built quartz imaging chamber (50 mm × 50 mm × 50 mm) with a transparent lid that could be screwed closed and sealed with a Gortex gasket. A pressure-release vent was incorporated into the lid that could be capped or connected to a balloon. Optical filters were mounted onto an optical breadboard between the ultraviolet projector and the quartz chamber, and between the quartz chamber and a camera for acquiring photographs. Experimenters wore goggles that blocked ultraviolet and blue light as both a safety precaution and as an optical filter. The effect of the filters on image quality was investigated using 515, 550 and 590 nm longpass filters between the quartz chamber and camera, and using a 240–395 nm bandpass filter between the ultraviolet projector and the quartz imaging chamber. The chamber was loaded with 5 mM **1** in dichloromethane and 1 p.p.m. triethylamine. A 25 × 25 pixel square was projected from the Pro4500 ultraviolet projector, and a 49 pixel green bar was projected from the Miroir 720p projector (Fig. 4). Figure 4a shows the image taken with no filter and Fig. 4b–d shows images using a 515, 550 and 590 nm longpass filter, respectively. Figure 4e–h shows the same images, but with the addition of a 240–395 nm bandpass filter between the Pro4500 ultraviolet projector and the Miroir 720p projector. In all cases, the inclusion of the ultraviolet bandpass filter reduced the background from the ultraviolet projector, which can be seen as the blue bar in the image without filters in Fig. 4a. Additionally, use of the longpass filters increased contrast of the projected image, resulting in qualitatively optimal contrast using the 550 nm longpass filter (Fig. 4g), which is supported by quantitative determinations of contrast (Supplementary Fig. 14).

### Characterization of imaging system

**Figure 5:**
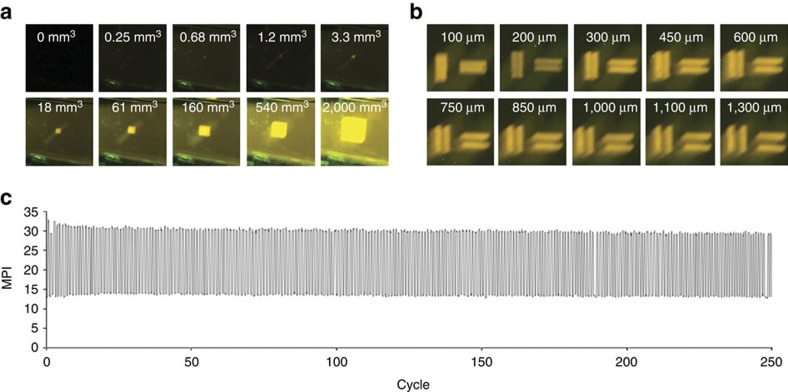
Characterization of the first-generation 3D Light PAD. (**a**) Images of voxels of increasing volume. (**b**) Images of bars with increasing separation in the vertical and horizontal direction. (**c**) Plot of MPI for on and off state of 5 mM **1** in CH_2_Cl_2_ containing 36 μM triethylamine versus cycle number. Images were generated using 2.0 mW cm^−2^ 525 nm light and 0.27 mW cm^−2^ 385 nm light.

Using this optimal optical filter configuration, the voxel size, resolution and repeatability of on-off cycles were characterized (Fig. 5). The smallest observable voxel was determined by systematically generating rectangular prisms and cubes of varying volumes (Supplementary Fig. 15). A selection of voxel sizes (Fig. 5a) revealed a minimum observable voxel volume to be between 0.25 and 0.68 mm^3^. As the voxel volume increases, the brightness increases (Supplementary Fig. 16a,b). The contrast also increases as the size of the ultraviolet projection increases, but reaches a maximum as the size of the green projection increases, eventually declining due to increased background (Supplementary Fig. 16c,d). The resolution was estimated by projecting horizontal and vertical bars and determining the minimum separation needed to differentiate the two bars (Fig. 5b). From these images, we determined a resolution between 100 and 200 μm in both the horizontal and vertical direction. The effect of the imaging depth on image brightness was evaluated with respect to both the Pro4500 ultraviolet projector (Supplementary Fig. 17a) and the Miroir 720p projector (Supplementary Fig. 17b). No clear difference in brightness was observed at different depths (Supplementary Fig. 17c,d), indicating that effects from absorption of the medium are minimal, given the current solvent composition and dimensions of the imaging chamber. In general, the images in this study were generated using 90° angles between the beams of the two projections, but the tolerance of smaller angles was also examined, with angles as low as 20° being physically tolerated in a typical set-up (Supplementary Fig. 18). The repeatability of the photoswitching was examined over 250 cycles, and generally shows excellent reproducibility of the MPI in photographs of both the ‘on’ image and ‘off’ image (Fig. 5c). This repeatability was also examined with different concentrations of triethylamine (Supplementary Fig. 19). While differences in minimum and maximum brightness were observed, the concentration of triethylamine had little effect on the repeatability. Finally, we examined the reusability of a single solution over multiple days by generating an image continuously for 1 h each day and evaluating the brightness and contrast at the end of each imaging session. Over this time frame, the contrast was consistent, with a slight increase in brightness over the 11-day period (Supplementary Fig. 20). In practice, the same solutions can effectively be used for image generation for weeks to months, with this gradual increase in brightness accumulating over time.

### 3D image generation

**Figure 6:**
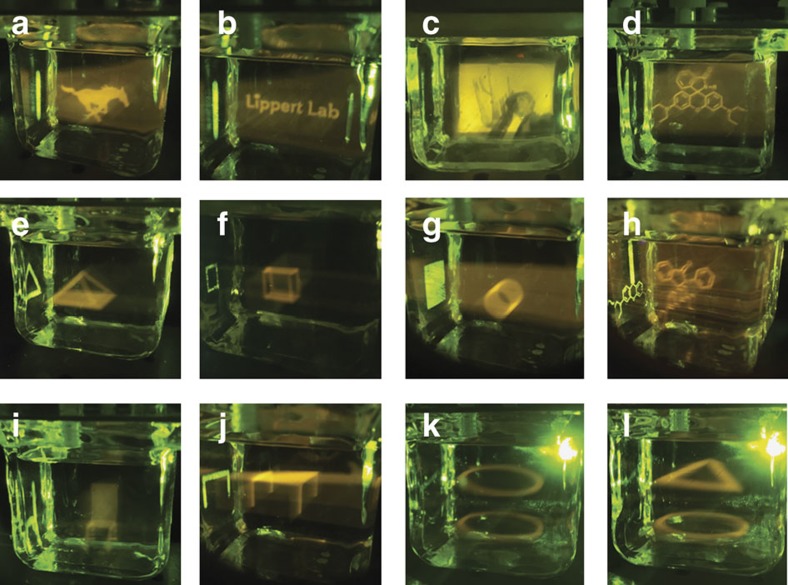
Representative images generated using the first-generation 3D Light PAD. (**a**) SMU Mustang, (**b**) ‘Lippert Lab’ text, (**c**) photograph of one of the authors next to a saguaro cactus, (**d**) *N*-phenyl spirolactam rhodamine B **1**, (**e**) pyramid, (**f**) cube, (**g**) cylinder, (**h**) *N*-phenyl spirolactam rhodamine **1** with 3D representation of stereochemistry, (**i**) chair, (**j**) table, (**k**) stacked circles, (**l**) stacked triangle and circle.

Having characterized the system parameters, images were generated using this first-generation 3D Light PAD (Fig. 6). First, high-resolution images were projected from the Pro4500 ultraviolet projector to fluoresce at a defined depth by projecting a green bar as a ‘screen’ of fluorescent activation (Fig. 6a–d and Supplementary Figs 21–24). Using this method, the SMU Mustang mascot (Fig. 6a), easily readable text (Fig. 6b), photographic images (Fig. 6c) and molecular structures (Fig. 6d) can be generated. In order to display more complex, spatially defined 3D images, the green Miroir 720p projector can be used to project a 2D visible light pattern to interact with a 2D ultraviolet pattern projected from the Pro4500 (Fig. 6e–g and Supplementary Figs 25–29). In Fig. 6e, a triangle is projected from both the green projector (Supplementary Fig. 25a) and the ultraviolet projector (Supplementary Fig. 25b) to generate a pyramid at the intersection. A similar strategy was used to generate images of a cube (Fig. 6f) and a cylinder (Fig. 6g). *N*-phenyl spirolactam rhodamine B **1** in the closed isomeric form adopts a perpendicular conformation at the spirolactam bond connection between the two rings. While it is difficult to accurately portray this 3D molecular structure in a traditional 2D display, this is easily rendered using a 3D Light PAD (Fig. 6h). Appropriate patterning can also be used to generate images of household objects such as a chair (Fig. 6i) or a table (Fig. 6j), providing a proof of principle for the utility of 3D Light PADs for computer-aided design. An outstanding challenge for beam-addressable 3D displays is the appearance of ‘ghost’ voxels caused by inevitable intersections of beams in undesired spaces^5^. As a preliminary strategy to address this issue, we introduce an approach where ultraviolet patterns are projected onto slices of green light to provide arbitrary 3D images without the excitation of ‘ghost’ voxels. This strategy gains resolution in the vertical dimension at the expense of resolution in one of the horizontal dimensions. Using this approach, arbitrary 3D patterns including stacked circles (Fig. 6k and Supplementary Fig. 30) and stacks of different-shaped objects (Fig. 6l and Supplementary Fig. 31) can be generated.

### 3D animations

Generating 3D animations must take into careful consideration the lifetime of the activated voxel. The discovery of base-tunable kinetics for the thermal fading reaction offers a strategy to increase the thermal relaxation rate, and we have acquired movies of a moving cube using increasing concentrations of triethylamine (Supplementary Movies 1–3). Using 1 p.p.m. triethylamine provides a bright cube, and animation proceeds smoothly when changing only the pattern from the green projector, but upon changing the ultraviolet pattern an after-image is seen, resulting in slowly fading ‘tracer’ images (Supplementary Movie 1). This can be improved by increasing the concentration of triethylamine to 5 p.p.m. (Supplementary Movie 2) or 10 p.p.m. triethylamine (Supplementary Movie 3). Considering that the lifetime of the fluorescence excited state is on the order of nanoseconds, we pursued a second animation strategy in which we projected a dynamic pattern of green light onto a ,static pattern of ultraviolet light. An animation of a running mustang was projected from the green Miroir 720p projector to intersect with a bar projected from the Pro4500 ultraviolet projector. This resulted in a movie of a horse running at 30 frames per second at a defined position in 3D space (Supplementary Movie 4). We anticipate that future optimization of the optical slicing strategy demonstrated in Fig. 6k,l will enable real-time animations of arbitrary 3D images.

## Discussion

This study describes the design, optimization, characterization and image generation using a 3D Light PAD. The display is enabled by fast photoactivation, an ∼415-fold increase in fluorescence emission upon photoactivation, the discovery of base-tunable thermal fading kinetics, and innovative use of DLP projector technology and optical filters. The display is inexpensive with a fabrication cost for this first-generation prototype of under $5,000, not including the cost of the two PCs used to control the projectors, which could conceivably be replaced by a single device (Supplementary Table 1). The current display attains a minimum viewable voxel volume of less than 0.68 mm^3^ and a resolution of 200 μm, with excellent repeatability in on-off cycles. High-resolution images can be projected at defined points in 3D space, and a wide range of 3D objects can be easily rendered, including a 3D molecular structure of the *N*-phenyl spirolactam rhodamine B **1** used in the display. An optical slicing strategy has been introduced as a method to overcome the problem of ‘ghost’ voxels and it is anticipated that improvements in photoactivatable dyes and DLP projector technologies will increase the impact of this strategy. 3D animations were constructed with a high refresh rate when projecting a dynamic green light projection onto a static ultraviolet photoactivated pattern. This system has no moving parts, uses low-power light sources, and is inexpensive and easy to implement, all important advantages over existing volumetric 3D displays. The 3D Light PAD can address a larger number of voxels with a smaller voxel size in comparison to other recently developed static volume volumetric 3D displays (Supplementary Table 2). It also compares favourably with regards to voxel size versus the leading swept-volume rotating screen displays developed by Favalora^8^ and Geng^9^, and we anticipate that future development will scale the system to larger volumes and a higher number of voxels without the need for high-speed projectors or moving parts. Outstanding challenges for this type of display include generation of a photoactivatable red-blue-green (RGB) dye set for a true-colour 3D display, overcoming the challenge of ‘ghost’ voxels, optimization of optical slicing or photoswitch kinetics for versatile real-time 3D animations, and implementation into a solid-state device. We anticipate that this 3D Light PAD strategy will be applied to the ever-growing family of photoactivatable molecules and DLP chip sets, providing exciting new opportunities for academic and industrial development of readily accessible volumetric 3D displays.

## Methods

### Spectroscopic methods

Absorbance spectra were obtained using a Beckman Coulter DU 800 Spectrophotometer (Fullerton, CA, USA). Fluorescence emission spectra were acquired using a Hitachi F-7000 Spectrophotometer (Hitachi, Tokyo, Japan). All spectra were acquired from samples in semi-micro fluorometer cells (Starna Scientific Ltd, Aascadero, CA, USA). The absorbance spectrum of the ‘off’ state was obtained from a sample of 50 μM **1** in dichloromethane. The sample was illuminated with 254 nm light (using the 150 W xenon lamp and monochromator equipped in the Hitachi F-7000, 20 nm excitation slits) for 5 min to obtain the absorbance spectrum of the ‘on’ state. The ‘off’ fluorescence emission spectrum was collected from a sample of 50 μM **1** in dichloromethane using an excitation wavelength of 556 nm. For the ‘on’ fluorescence emission spectrum, a sample of 50 μM **1** in dichloromethane was irradiated with 254 nm light (using the 150 W xenon lamp and monochromator equipped with the Hitachi F-7000, 20 nm excitation slits) for 5 min before the emission spectrum was collected using an excitation wavelength of 556 nm. The kinetics of thermal fading of 5 mM **1** were measured in dichloromethane. The sample was illuminated with 315 nm light (using the 150 W xenon lamp and monochromator equipped with the Hitachi F-7000, 20 nm excitation slits) for 1 min and then transferred to the DU 800 spectrophotometer, and absorbance spectra were acquired at regular intervals. The peak absorbance value was plotted versus time and the plot was fit to a single exponential using Microsoft Excel. Experiments were performed at ambient temperature (20–23 °C). Irradiation power was measured using a ThorLabs (Newton, NJ, USA) Energy Console Meter (PM100D) equipped with a Photodiode Power Sensor, 200–1,100 nm (S120VC). More details can be found in the Supplementary Methods.

### 3D imaging apparatus

Images were acquired using a smartphone camera. Preliminary images in Fig. 1e were acquired using 5 mM **1** in dichloromethane dissolved in a 4-dram vial, a 5 mW 405 nm laser pointer (Carolina Biological Supply Company, Burlington, NC, USA) and a Logitech Professional Presenter R800 equipped with a 5 mW 532 nm laser pointer (Logitech, Newark, CA, USA). The beams were aimed towards each other and a photograph was taken of the voxel of light emitted at the intersection. All other images were acquired in the apparatus described in Fig. 3. Optical filters, filter mounts, 18" × 24" × 1/2" aluminium breadboard, and posts were purchased from Thorlabs. Ultraviolet structured light was generated with a Pro4500 Wintech Production Ready Optical Engine equipped with a 385 and 525 nm LED (Wintech Digital Systems Technology Corp., Carlsbad, CA, USA). The projector was controlled using the Lightcrafter 4500 GUI (Texas Instruments, Dallas, TX, USA) set to video mode according to the manufacturer's instructions. LED driver control was set so that the red/green LED current was set to 0 and the blue LED current was set to the blue LED current value of 150. Green structured light was generated with a Miroir 720p picoprojector. Images were generated using solutions of **1** in 4-dram glass vials, Fisherbrand Clear Straight-Sided Jars with PTFE-Faced PE-Lined Caps, or a custom 50 mm × 50 mm × 50 mm quartz tank equipped with a screw-tightened clear quartz lid, Goretex gasket and a capped pressure-release vent on the lid (Imtec Acculine, Fremont, CA, USA).

### Thermal fading kinetics in the presence of triethylamine

Solutions of 1 p.p.m. (7.2 μM), 5 p.p.m. (36 μM) or 15 p.p.m. (108 μM) triethylamine and 5 mM **1** in dichloromethane were prepared in a 4-dram vial. For each test, the vial was placed at the focal length (184 mm) of the Pro4500 ultraviolet projector, and the Miroir was placed 101 mm away from the centre of the imaging chamber. A square (25 × 25 pixels) was projected from the ultraviolet projector against a green plane (width of 49 pixels) for 4–10 s, and then the ultraviolet projector was switched to a blank slide for 4–10 s to allow the voxel to turn off. This cycle was repeated 3 times and recorded by a video from the stock smartphone camera app. The video was cropped for each cycle from the QuickTime application. Each cropped video was converted into an image sequence using MPEG Streamclip (version 1.9.2) application. The image sequences were then opened in ImageJ and the MPI was measured and graphed against time. Plots were fitted in Mathematica using Equation1, where *I* is the MPI as a function of time, *B* is the background intensity, *A* represents the coefficient at maximum emission, *k* is the observed rate constant and *t* is the time in seconds:


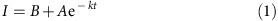


More details can be found in the Supplementary Methods.

### Evaluation of optical filters

Solutions of 5 mM **1** in dichloromethane were prepared with 1 p.p.m. (7.2 μM) triethylamine. The imaging chamber was placed at the focal length (184 mm) of the ultraviolet projector and the Miroir 720p projector was placed 101 mm away from the centre of the imaging chamber. A square (25 × 25 pixels) was projected from the ultraviolet projector against a green plane (width of 49 pixels) for 10 s before images were taken. Pictures were taken with no filter, a 515 nm longpass filter, a 550 nm longpass filter and a 590 nm longpass filter mounted in front of the camera. Images were captured with and without a 240–395 nm bandpass filter mounted in front of the ultraviolet projector for each longpass filter. MPI of a selected region of interest was measured using ImageJ. More details can be found in the Supplementary Methods.

### Evaluation of voxel size and resolution

A solution of 5 mM **1** in dichloromethane containing 1 p.p.m. (7.2 μM) triethylamine was prepared. Voxels were generated by projecting green bars of varying widths from the Miroir 720p projector placed 115 mm away from the centre of the imaging chamber and projecting a square of varying area from the Pro4500 ultraviolet projector placed at 184 mm from the centre of the imaging chamber. These images were projected against a green plane for 5 s before images were taken. A 240–395 nm bandpass filter was mounted in front of the ultraviolet projector, and a 550 nm filter was mounted in front of the camera. For resolution experiments, the imaging chamber was placed at the focal length (184 mm) of the ultraviolet projector and the Miroir 720p projector was placed 101 mm away from the centre of the imaging chamber. Two rectangles with no outline in both the horizontal and the vertical direction were spaced to be 1, 2, 3, 4, 5, 6, 7, 8, 9 and 10 pixels apart. Distances in μm were determined using the throw ratios, resolution, and projected pixel size indicated in the manufacturer specifications and the measured distance of the ultraviolet projector from the image. These measurements were confirmed using manual measurements of projections. More details can be found in the Supplementary Methods.

### Evaluation of repeatability

A solution of 5 mM **1** in dichloromethane was prepared with 2 p.p.m. (14.4 μM), 5 p.p.m. (36 μM) or 15 p.p.m. (108 μM) of triethylamine. The imaging chamber was placed at the focal length (184 mm) of the ultraviolet projector and the Miroir 720p projector was placed 101 mm away from the centre of the imaging chamber. A 240–395 nm bandpass filter was mounted in front of the ultraviolet projector, and a 550 nm filter was mounted in front of the camera. A square (25 × 25 pixels) was projected from the ultraviolet projector against a green plane (width of 64 pixels) for 10 s, and then the ultraviolet projector was switched to a blank slide for 10 s to allow the voxel to turn off. This cycle was repeated for 75–250 cycles. The smartphone camera application was used to automatically take pictures every 10 s, capturing the ‘on’ state and the ‘off’ state for each cycle. More details can be found in the Supplementary Methods. MPI was measured using ImageJ.

### General methods for generation of images and animations

A solution of 5 mM **1** was prepared with 1 p.p.m. (7.2 μM) of triethylamine. The imaging chamber was placed at the focal length (184 mm) of the ultraviolet projector and the Miroir 720p projector was placed 101 mm away from the centre of the imaging chamber. In some cases, the concentrations of triethylamine and the distances and tilts of the projectors were empirically adjusted for optimal image quality; see Supplementary Figs 21–31 for more details. A 240–395 nm bandpass filter was mounted in front of the ultraviolet projector, and the 550 nm filter was mounted in front of the camera. Static images were projected from the Wintech Pro4500 ultraviolet projector and the Miroir 720p projector for 5 to 10 s before photographs were captured. Generally, images were generated using 2.0 mW cm^−2^ 525 nm light and 0.27 mW cm^−2^ 385 nm light, with small deviations due to projector tilt and distance. More details can be found in the Supplementary Methods.

### Data availability

Data supporting the findings in this study are available within the article and its supplementary information files and from the corresponding author upon reasonable request.

## Additional information

**How to cite this article:** Patel, S. K. *et al*. A volumetric three-dimensional digital light photoactivatable dye display. *Nat. Commun.* **8,** 15239 doi: 10.1038/ncomms15239 (2017).

**Publisher’s note:** Springer Nature remains neutral with regard to jurisdictional claims in published maps and institutional affiliations.

## Supplementary Material

Supplementary Information Supplementary figures, supplementary tables, supplementary methods and supplementary references.

Supplementary movie 1 Three-dimensional animation of a cube moving in the x-, y-, and z- planes. Animation projected in 5 mM 1 solution containing 1 ppm triethylamine. The Pro4500 UV projector was placed 184 mm away from the center of the imaging chamber (at the focal length of the projector) and the Miroir 720p projector was placed 177 mm away from the center of the imaging chamber. A 240-395 nm bandpass filter was mounted in front of the UV projector, and a 550 nmlongpass filter was mounted in front of a smartphone camera.

Supplementary movie 2 Three-dimensional animation of a cube moving in the x-, y-, and z- planes in the same conditions as Supplementary Movie 1 but containing 5 ppm triethylamine rather than 1 ppm.

Supplementary movie 3 Three-dimensional animation of a cube moving in the x-, y-, and z- planes in the same conditions as Supplementary Movie 1 but containing 10 ppm triethylamine rather than 1 ppm.

Supplementary movie 4 Two-dimensional animation of a horse running. Animation projected in a solution of 5 mM 1 containing 0.8 ppm triethylamine. Each image of the horse was projected from the Miroir 720p projector placed 228 mm away from the center of the imaging chamber and the Pro4500 UV projector was placed 184 mm away from the center of the chamber. The images were projected against a UV plane that was the width of 16 pixels. A 240-395 nm bandpass filter was mounted in front of the Pro4500 UV projector, and a 550 nm long pass filter was mounted in front of a smartphone camera.
