# Supplementary material for: A volumetric three-dimensional digital light photoactivatable dye display
Source: Nat Commun. 2017 Jul 11;8:15239. doi: 10.1038/ncomms15239 (PMC5508202; doi:10.1038/ncomms15239)
Supplement: Supplementary Information — Supplementary figures, supplementary tables, supplementary methods and supplementary references. [file ncomms15239-s1.pdf]

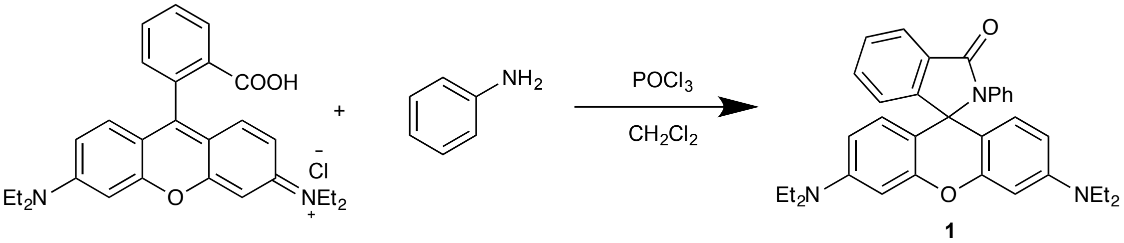

Supplementary Figure 1. Synthesis of **1**.

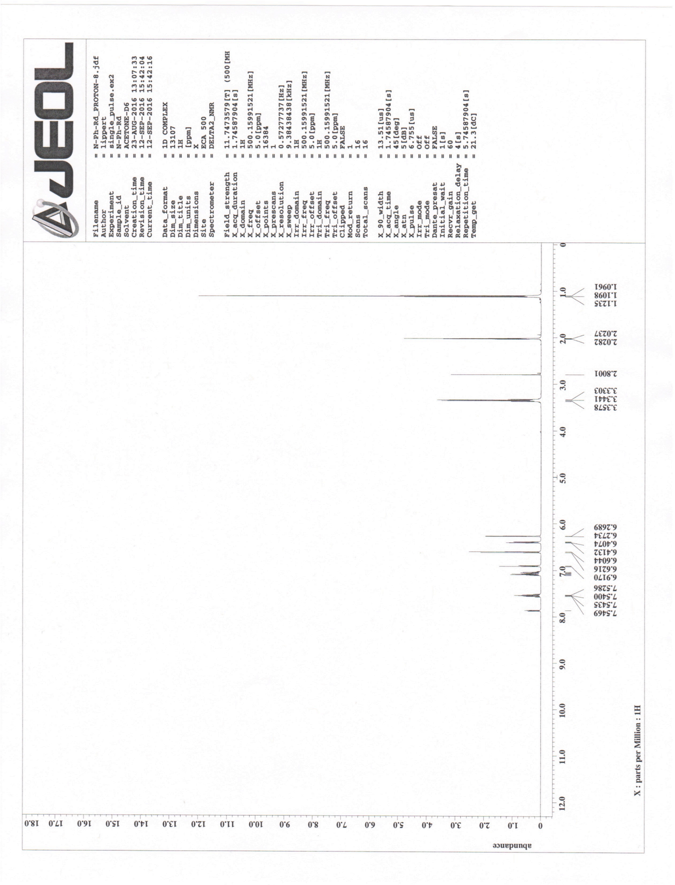

Supplementary Figure 2. ^{1}H NMR spectrum of **1** in $(CD_3)_2CO$.

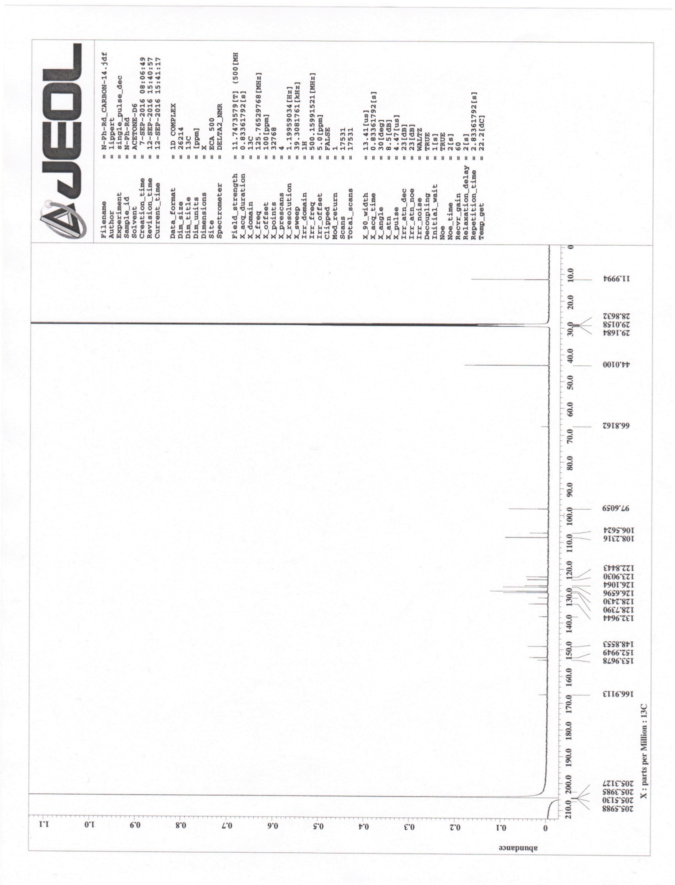

Supplementary Figure 3. ^{13}C NMR spectrum of **1** in $(CD_3)_2CO$.

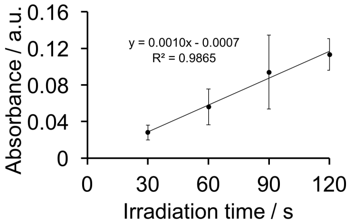

Supplementary Figure 4. Absorbance in arbitrary units (a.u.) at 556 nm after varying intervals of illumination of 5 mM **1** in CH_2Cl_2 with 1.0 mW/cm^2 315 nm light. Error bars are ± S.D.

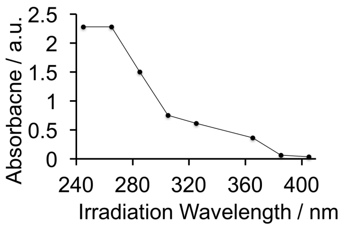

Supplementary Figure 5. Absorbance in arbitrary units (a.u.) at 556 nm of 5 mM **1** in CH_2Cl_2 after 1 minute of irradiation of light of varying wavelengths.

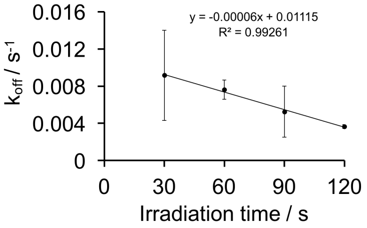

Supplementary Figure 6. Rate of thermal fading of 5 mM **1** in CH_2Cl_2 after varying illumination time with 1.0 mW/cm^2 315 nm. Error bars are ± S.D.

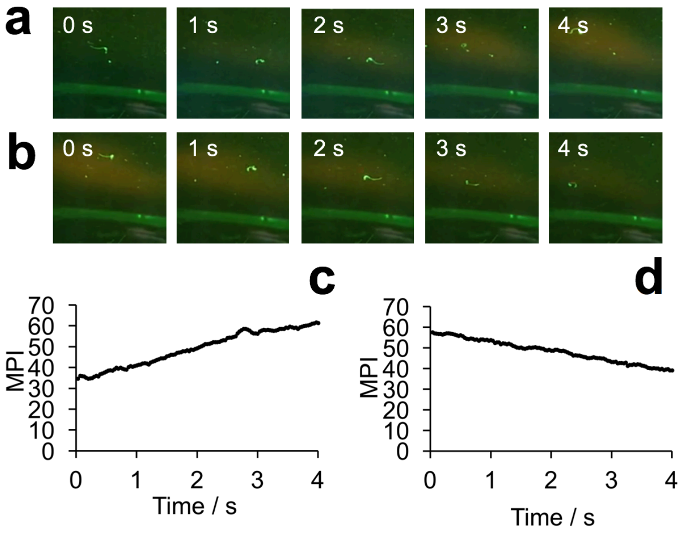

Supplementary Figure 7. Thermal fading in the presence of 1 ppm (7.2 μM) triethylamine. (a) Images of 5 mM **1** in CH_2Cl_2 and 1 ppm triethylamine (7.2 μM) illuminated with 0.40 mW/cm^2 385 nm and 1.7 mW/cm^2 525 nm light for 0, 1, 2, 3, and 4 seconds. (b) Images of 5 mM **1** in CH_2Cl_2 and 1 ppm triethylamine (7.2 μM) after illumination with 0.40 mW/cm^2 385 nm and 1.7 mW/cm^2 525 nm light for 4 seconds, and then removing the 0.40 mW/cm^2 385 nm illumination for 0, 1, 2, 3, and 4 seconds. (c) Plot of mean pixel intensity (MPI) of illuminated cross section versus time for the photoactivation experiment shown in (a). (d) Plot of mean pixel intensity (MPI) of illuminated cross section versus time for the thermal fading experiment shown in (b).

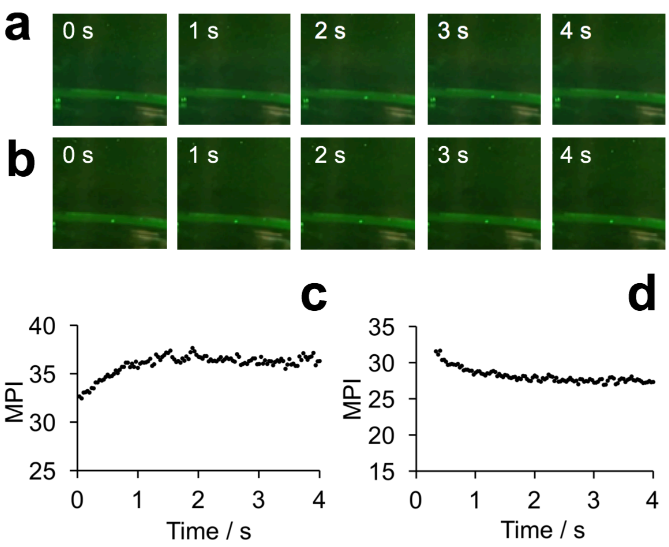

Supplementary Figure 8. Thermal fading in the presence of 15 ppm (108 μM) triethylamine. (a) Images of 5 mM **1** in CH_2Cl_2 and 15 ppm triethylamine (108 μM) illuminated with 0.40 mW/cm^2 385 nm and 1.7 mW/cm^2 525 nm light for 0, 1, 2, 3, and 4 seconds. (b) Images of 5 mM **1** in CH_2Cl_2 and 15 ppm triethylamine (108 μM) after illumination with 0.40 mW/cm^2 385 nm and 1.7 mW/cm^2 525 nm light for 4 seconds, and then removing the 0.40 mW/cm^2 385 nm illumination for 0, 1, 2, 3, and 4 seconds. (c) Plot of mean pixel intensity (MPI) of illuminated cross section versus time for the photoactivation experiment shown in (a). (d) Plot of mean pixel intensity (MPI) of illuminated cross section versus time for the thermal fading experiment shown in (b).

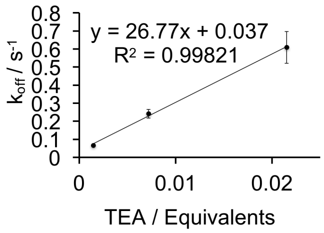

Supplementary Figure 9. Dependence of thermal fading kinetics on triethylamine. Thermal fading rate is plotted versus the equivalents of triethylamine in comparison to **1**. Data collected with 5 mM **1** and 7.2 μM, 36 μM, or 108 μM triethylamine in CH_2Cl_2. Error bars are ± S.D.

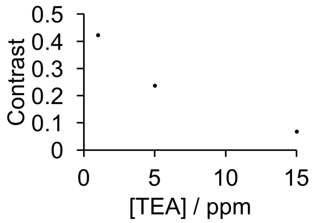

Supplementary Figure 10. Contrast as determined by Supplementary Equation (1) of images projected in 5 mM **1** in CH_2Cl_2 containing 1, 5, and 15 ppm triethylamine.

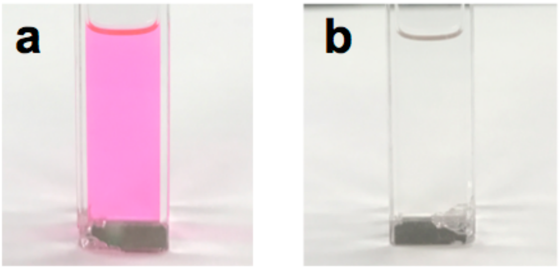

Supplementary Figure 11. Decoloration with diisopropylethylamine. Image of 5 mM **1** in CH_2Cl_2 after irradiating with 0.26 mW/cm^2 254 nm light for 5 minutes. (b) Image of 5 mM **1** in CH_2Cl_2 after irradiating with 0.26 mW/cm^2 254 nm light for 5 minutes, followed by addition of 1 drop of diisopropylethylamine.

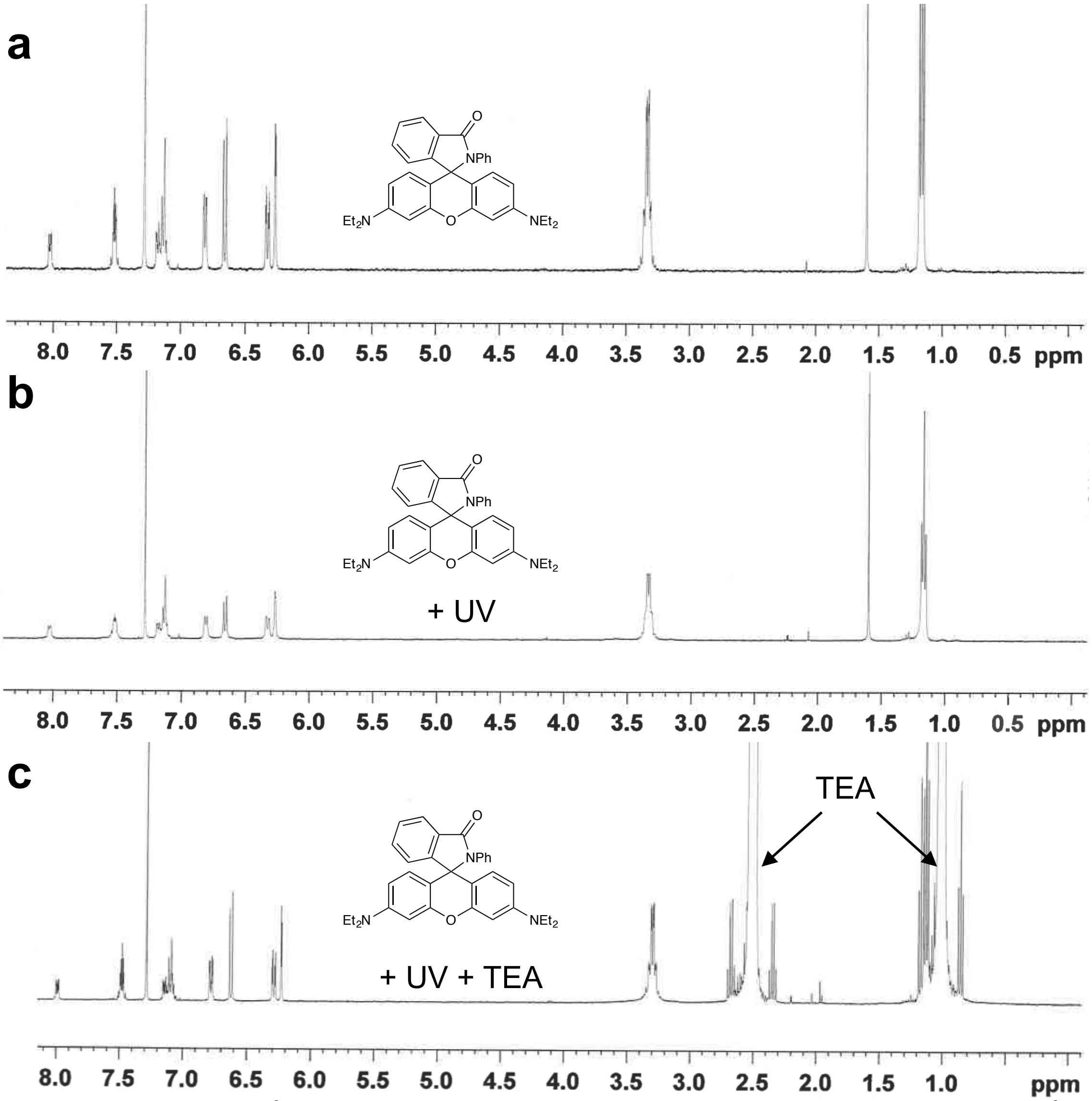

Supplementary Figure 12. ^{1}H NMR studies of the effect of triethylamine on thermal fading. (a) ^{1}H NMR spectra (500 MHz) of 5 mM **1** in $CDCl_3$. (b) ^{1}H NMR spectra (500 MHz) of 5 mM **1** in $CDCl_3$ after irradiating with 0.26 mW/cm^2 254 nm light for 5 minutes. (c) ^{1}H NMR spectra (500 MHz) of 5 mM **1** in $CDCl_3$ after irradiating with 0.26 mW/cm^2 254 nm light for 5 minutes, followed by addition of excess triethylamine.

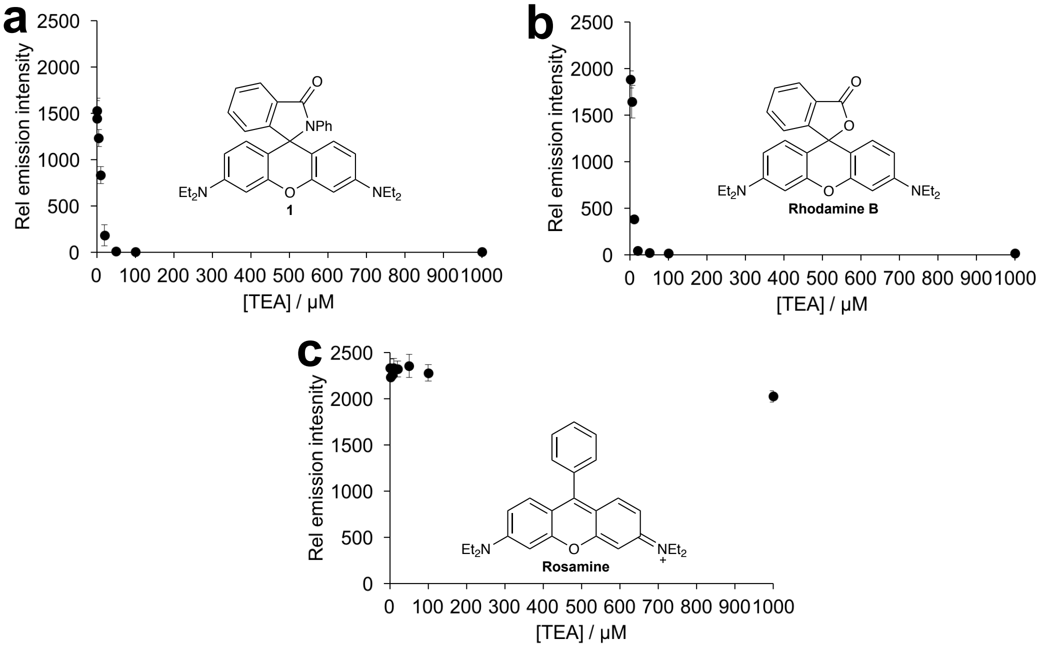

Supplementary Figure 13. Fluorescence emission of 5 mM (a) **1** after irradiating with 0.26 mW/cm^2 254 nm light for 5 minutes, (b) rhodamine B, or (c) rosamine and 0–1000 μM triethylamine in CH_2Cl_2.

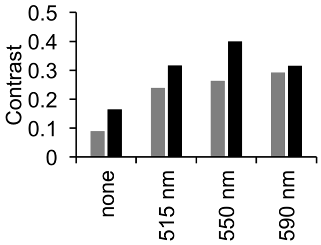

Supplementary Figure 14. Effect of filters on contrast. Contrast of voxels using no filter, 515 nm, 550 nm, or 590 nm longpass filter mounted in front of the camera without (gray bars) and with (black bars) a 240–395 nm bandpass filter mounted in front of the Pro4500 UV projector.

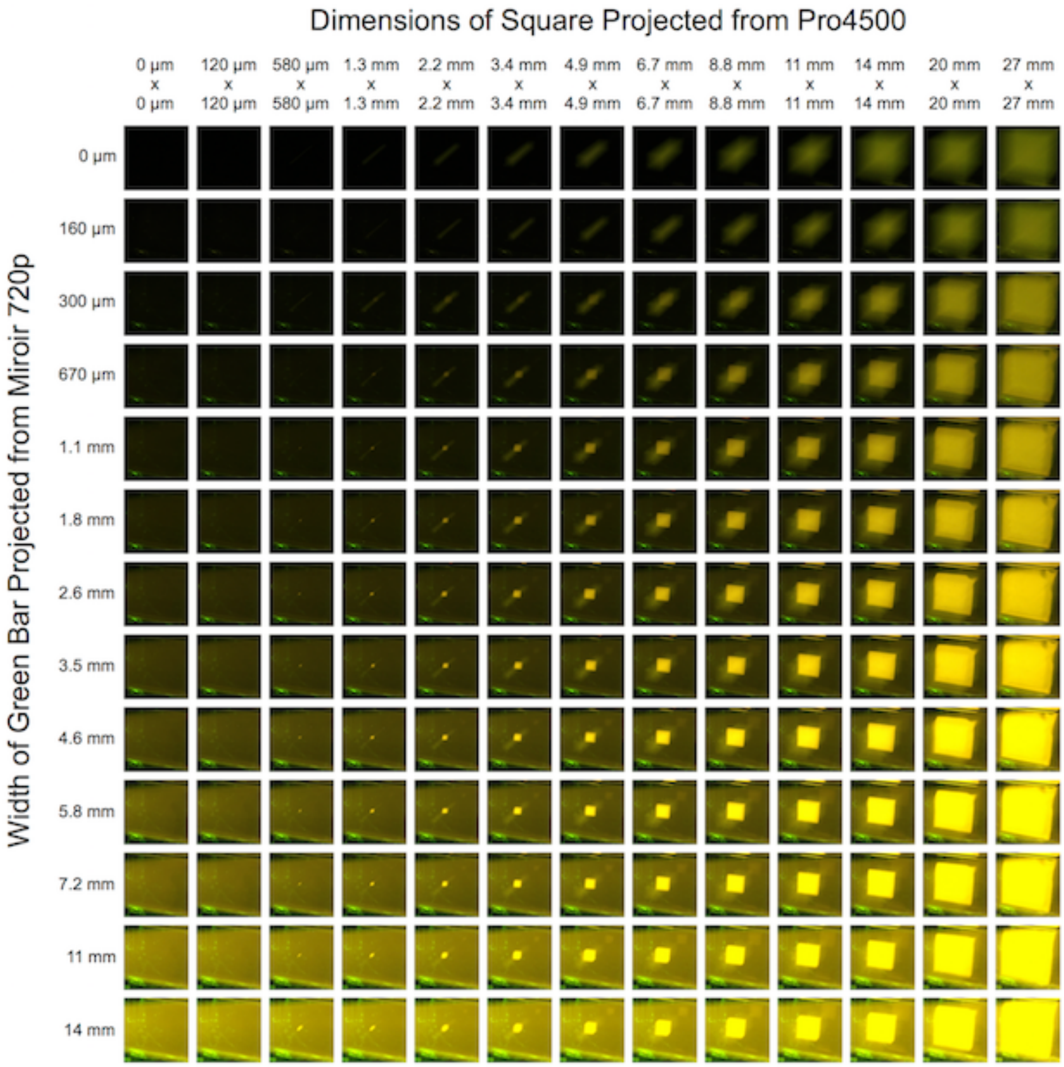

Supplementary Figure 15. Characterization of voxel size. Images formed from squares of side lengths of 0 µm, 120 µm, 580 µm, 1.3 mm, 2.2 mm, 3.4 mm, 4.9 mm, 6.7 mm, 8.8 mm, 11 mm, 14 mm, 20 mm, and 27 mm projected from the Pro4500 UV projector and plane widths of 0 µm, 160 µm, 300 µm, 670 µm, 1.1 mm, 1.8 mm, 2.6 mm, 3.5 mm, 4.6 mm, 5.8 mm, 7.2 mm, 11 mm, and 14 mm projected from the Miroir 720p projector. Data was collected using 1.9 mW/cm^2 525 nm light and 0.27 mW/cm^2 385 nm light.

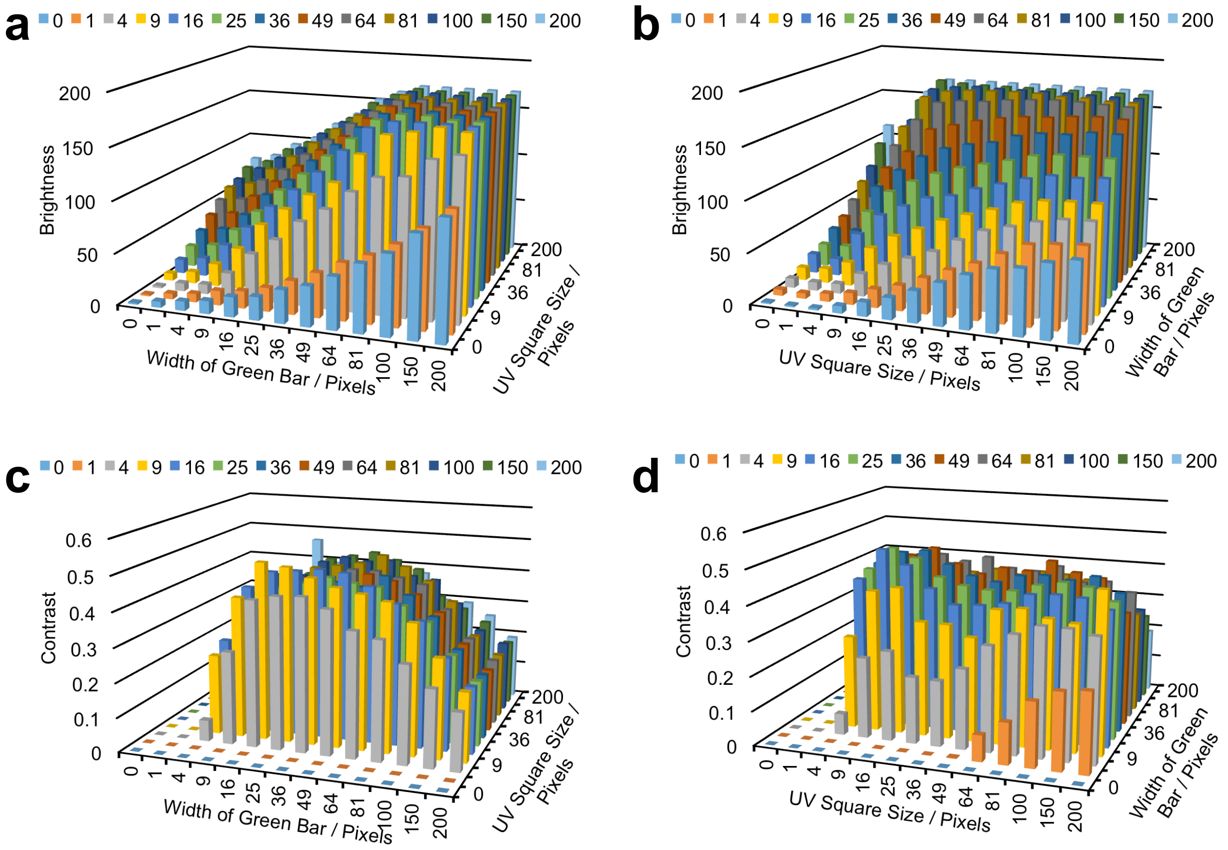

Supplementary Figure 16. Brightness and contrast measurements for varying square sizes. (a)–(b) Three-dimensional plot of brightness versus voxel size. The graph in (b) is the same graph as (a), but rotated 90 degrees. (c)–(d) Three-dimensional plot of contrast versus voxel size. The graph in (d) is the same graph as (c), but rotated 90 degrees.

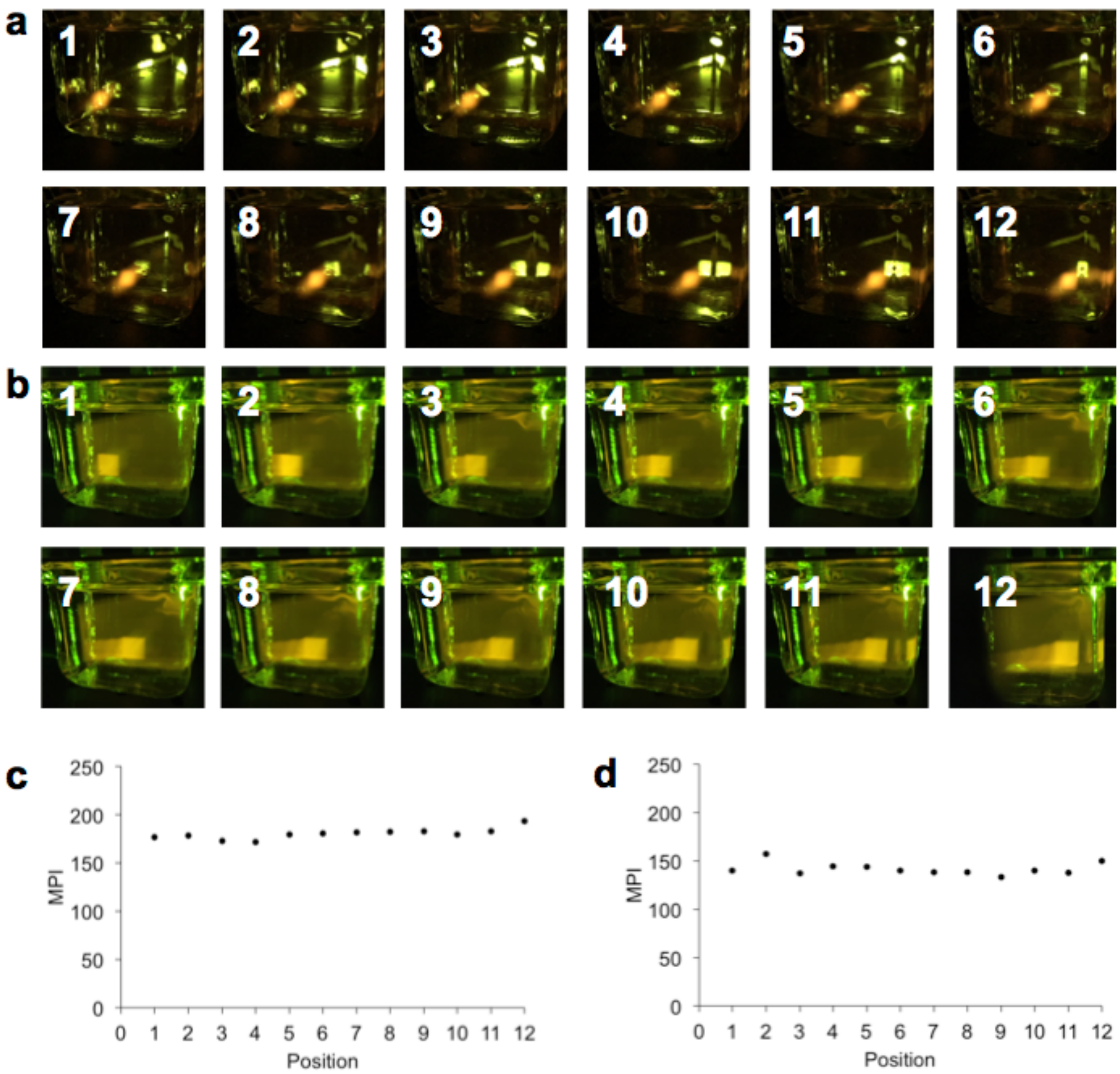

Supplementary Figure 17. Evaluation of the effect of imaging depth on brightness. (a) Images of a green square projected onto a UV beam at different depths. Position **1** is farthest from and position **12** is closest to the Pro4500 UV projector. (b) Images of a UV square projected onto a green beam at different depths. **1** is farthest from and **12** is closest to the Miroir 720p projector. (c) Quantification of the mean pixel intensity (MPI) of the projected squares in (a). (d) Quantification of the mean pixel intensity (MPI) of the projected squares in (b). Data was collected using 2.0 mW/cm^2 525 nm light and 0.27 mW/cm^2 385 nm light.

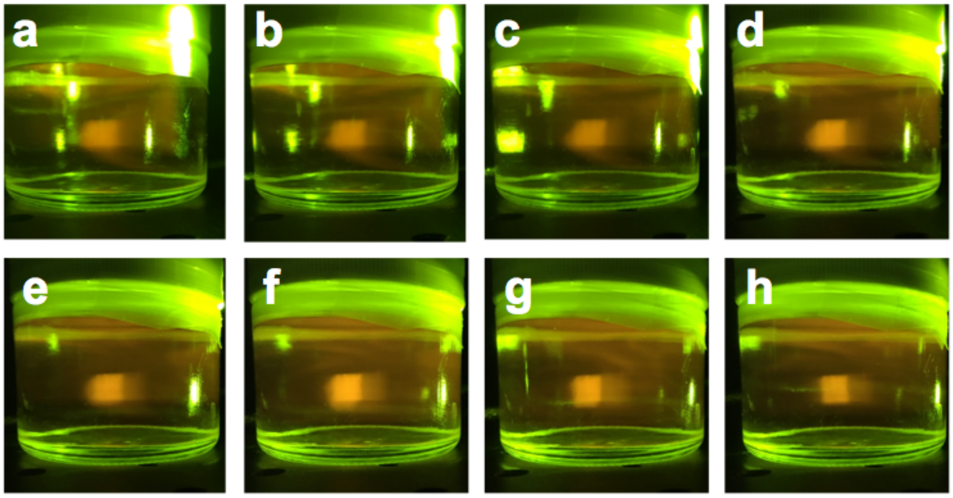

Supplementary Figure 18. Images generated by aiming a square from the Pro4500 UV projector and a green bar from the Miroir 720p projector with the beams of the two projectors angled at (a) 20º, (b) 30º, (c), 40º, (d) 50º, (e) 60º, (f) 70º, (g) 80º, and (h) 90º. Data was collected using 2.0 mW/cm^2 525 nm light and 0.27 mW/cm^2 385 nm light.

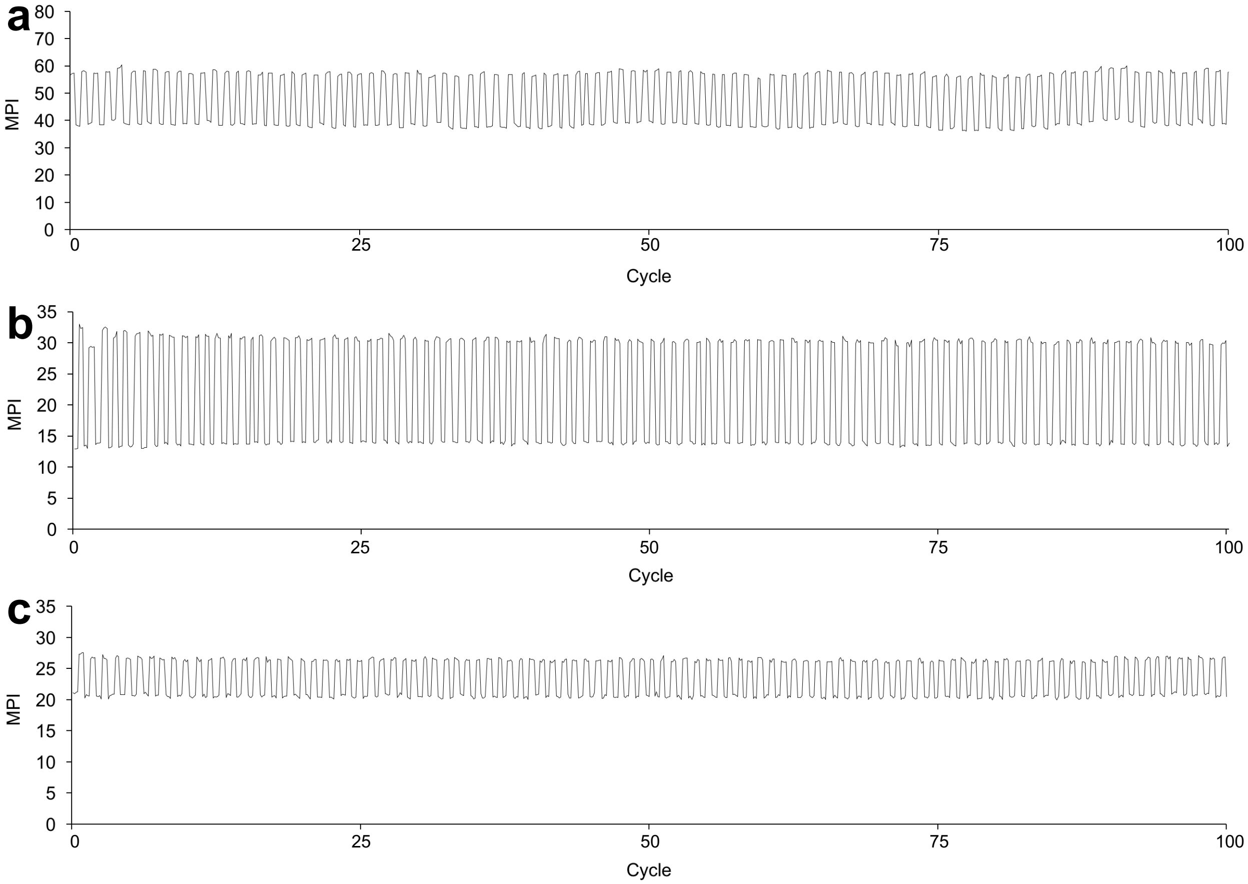

Supplementary Figure 19. Repeatability with variable concentrations of triethylamine. A square was projected from the Pro4500 UV projector onto a bar from the Miroir 720p green projector. The square was switched on and off over 100 cycles. The solutions consisted of 5 mM **1** in CH_2Cl_2 containing (a) 14.4 µM, (b) 36 µM, or (c) 108 µM triethylamine. Data was collected using 2.0 mW/cm^2 525 nm light and 0.27 mW/cm^2 385 nm light.

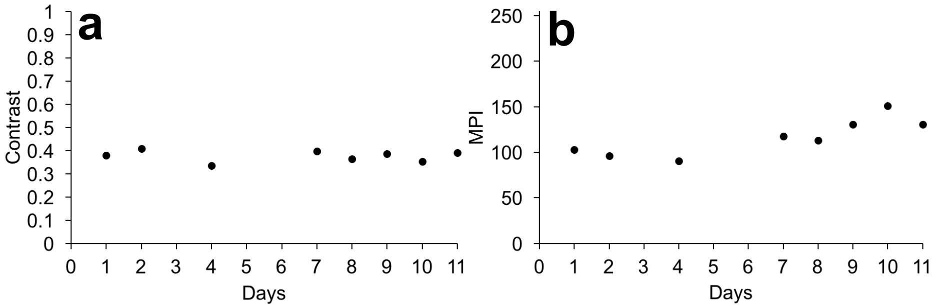

Supplementary Figure 20. Evaluation of the (a) contrast and (b) brightness (mean pixel intensity) of images of a square after one hour of continuous operation repeated with the same solution on subsequent days. Data was collected using 2.0 mW/cm^2 525 nm light and 0.27 mW/cm^2 385 nm light.

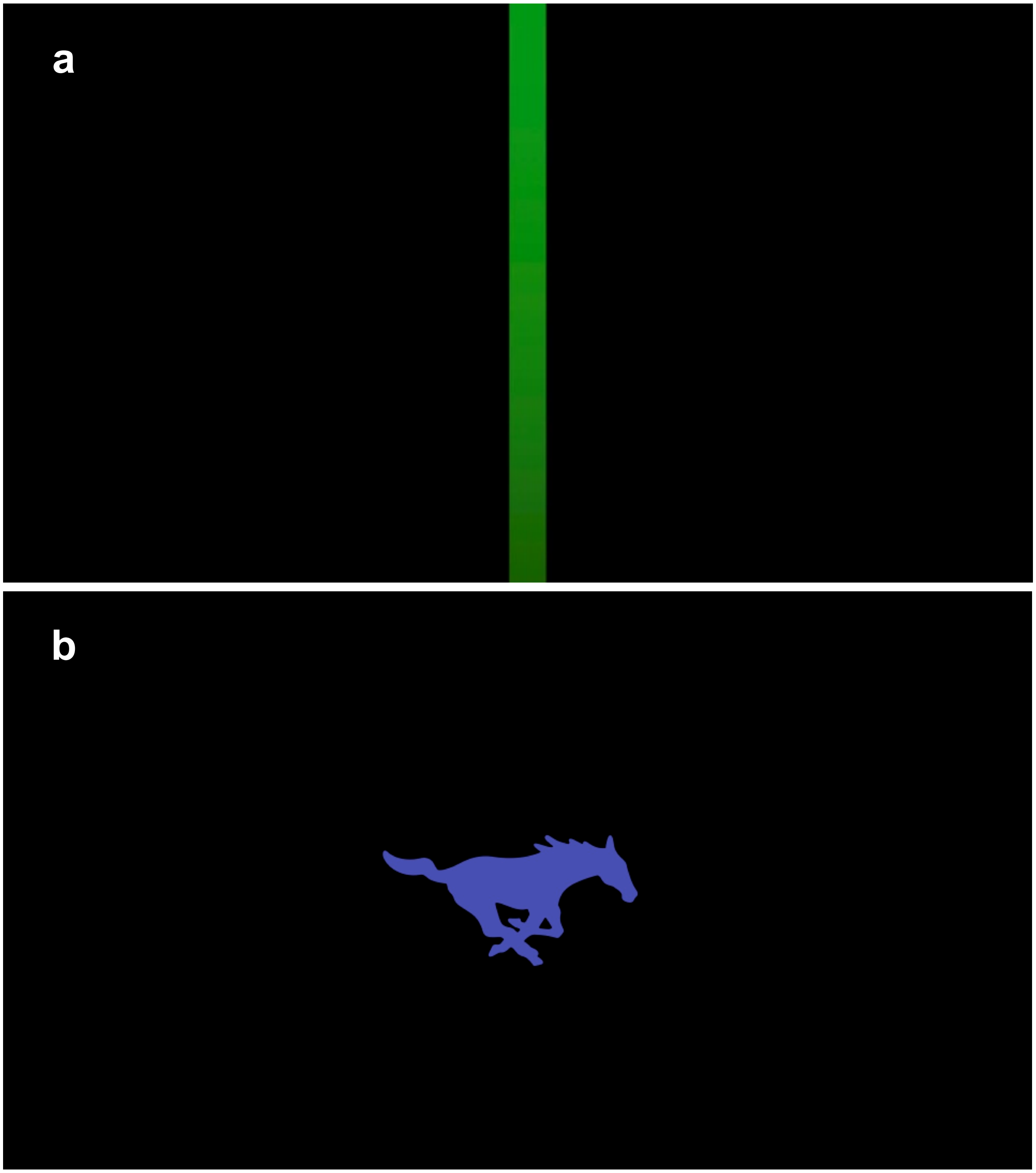

Supplementary Figure 21. Image projection for the Southern Methodist University 'Mustang' mascot. Images were projected into a 5 mM solution of **1** in CH_2Cl_2 with 0.8 ppm triethylamine. The Pro4500 UV projector was placed at focal length, which was 184 mm away from the center of the imaging chamber. The Miroir 720p projector was placed 101 mm away from the center of the imaging chamber. A 240–395 nm bandpass filter was mounted in front of the Pro4500 UV projector, and a 550 nm filter was mounted in front of the camera. The image was projected from the UV projector against a plane width of 64 pixels. (a) PowerPoint slide projected from the Miroir 720p projector. (b) PowerPoint slide projected from the Wintech Pro4500 UV projector.

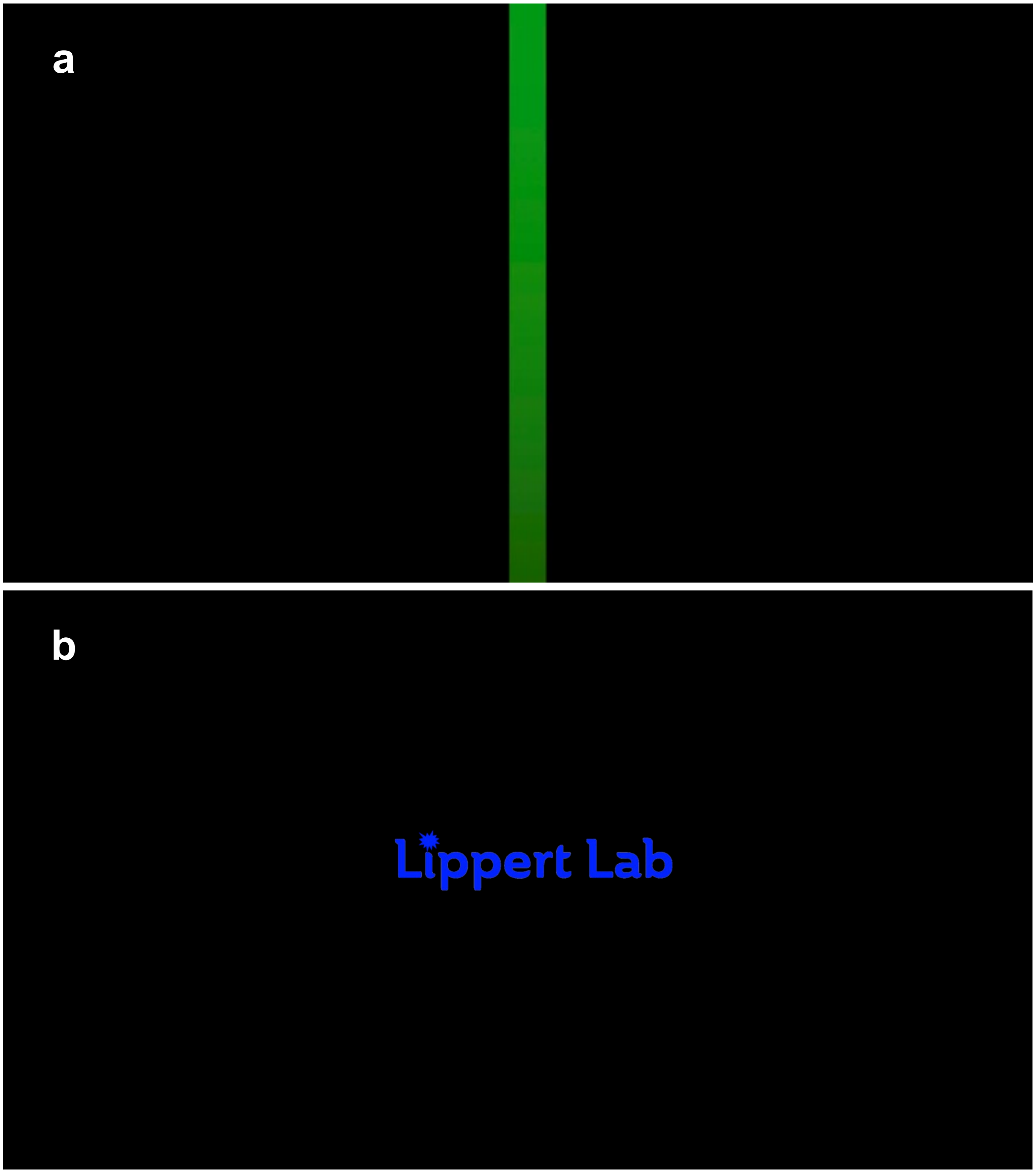

Supplementary Figure 22. Image projection for the "Lippert Lab" text**.** Images were projected into a 5 mM solution of **1** in CH_2Cl_2 with 0.8 ppm triethylamine. The Pro4500 UV projector was placed at focal length, which was 184 mm away from the center of the imaging chamber. The Miroir 720p projector placed 101 mm away from the center of the imaging chamber. A 240–395 nm bandpass filter was mounted in front of the Pro4500 UV projector, and a 550 nm filter was mounted in front of the camera. The image was projected from the Pro4500 UV projector against a plane width of 64 pixels. (a) PowerPoint slide projected from the Miroir projector. (b) PowerPoint slide projected from the Wintech Pro4500 UV projector.

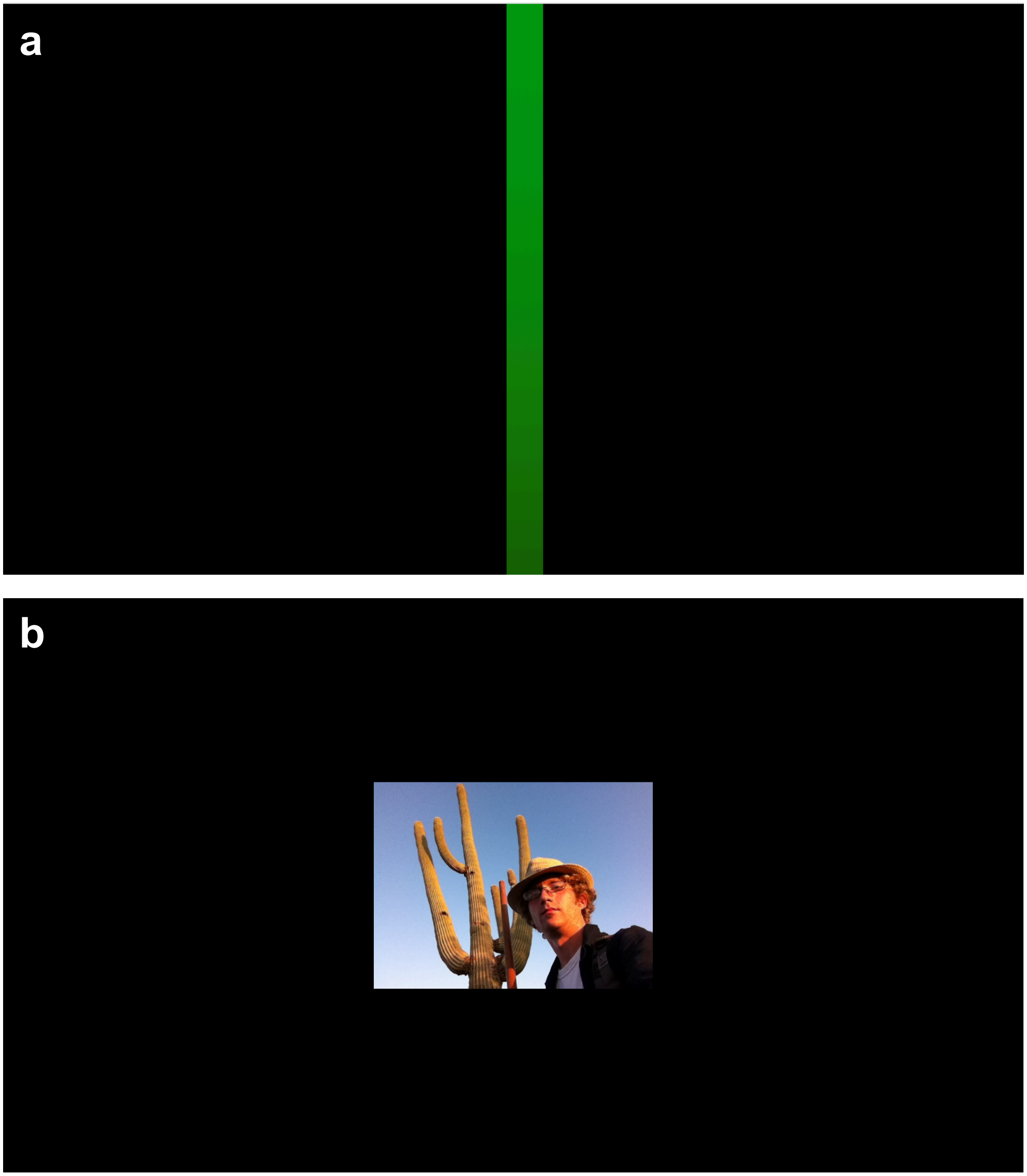

Supplementary Figure 23. Image projection for the photograph of the author next to a saguaro cactus. Images were projected into a 5 mM solution of **1** in CH_2Cl_2 with 0.8 ppm triethylamine. The Pro4500 UV projector was placed at focal length, which was 184 mm away from the center of the imaging chamber. The Miroir projector placed 101 mm away from the center of the imaging chamber. A 240–395 nm bandpass filter was mounted in front of the UV projector, and a 550 nm filter was mounted in front of the camera. The two-dimensional image was projected from the UV projector against a plane width of 64 pixels. (a) PowerPoint slide projected from the Miroir projector. (b) PowerPoint slide projected from the Wintech 4500 UV projector.

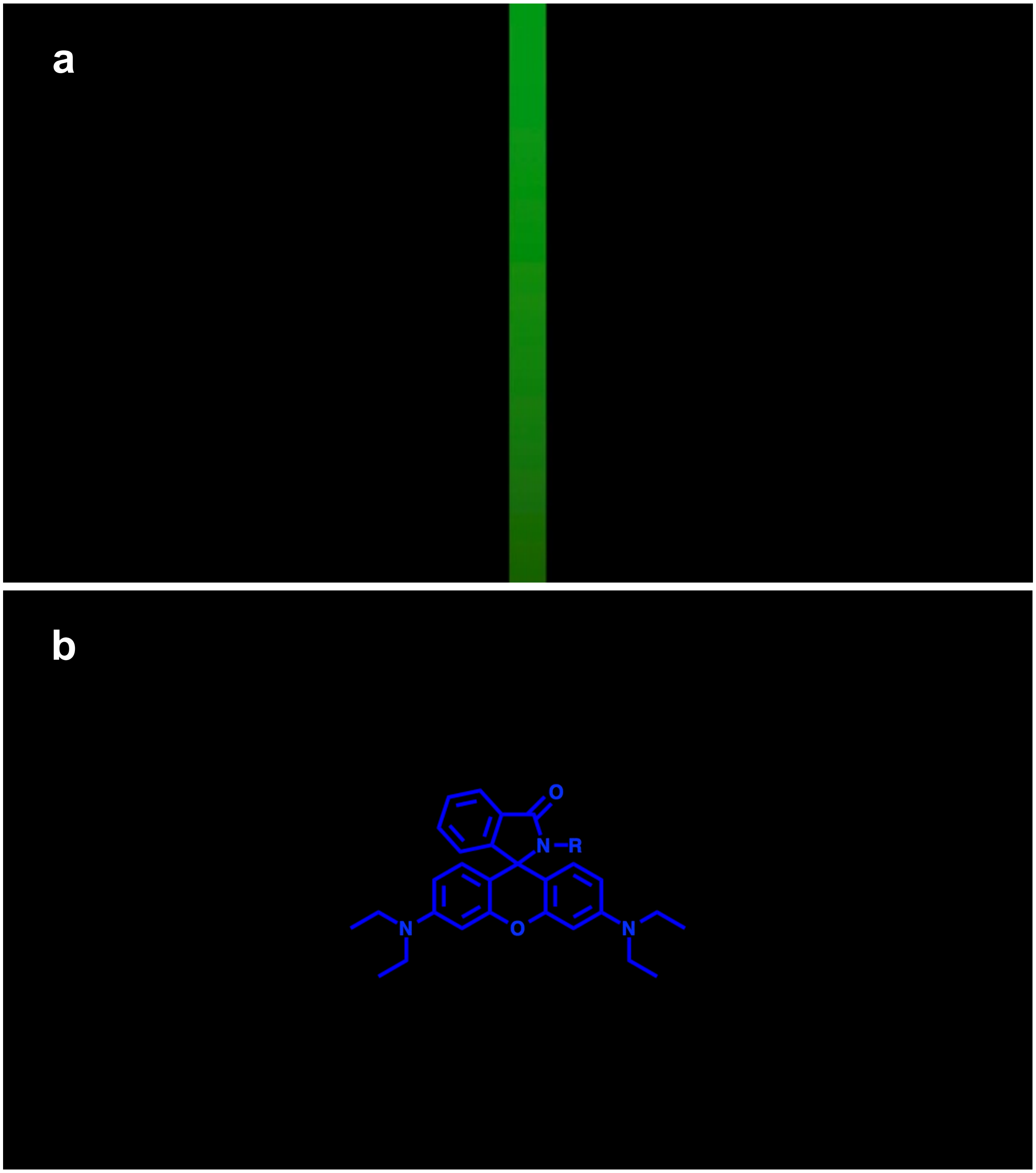

Supplementary Figure 24. Image projection for the dye structure of **1**. Images were projected into a 5 mM solution of **1** in CH_2Cl_2 with 0.8 ppm triethylamine. The Pro4500 UV projector was placed at focal length, which was 184 mm away from the center of the imaging chamber. The Miroir 720p projector was placed 101 mm away from the center of the imaging chamber. A 240–395 nm bandpass filter was mounted in front of the Pro4500 UV projector, and a 550 nm filter was mounted in front of the camera. The image was projected from the Pro4500 UV projector against a plane width of 64 pixels. (a) PowerPoint slide projected from the Miroir 720p projector. (b) PowerPoint slide projected from the Wintech Pro4500 UV projector.

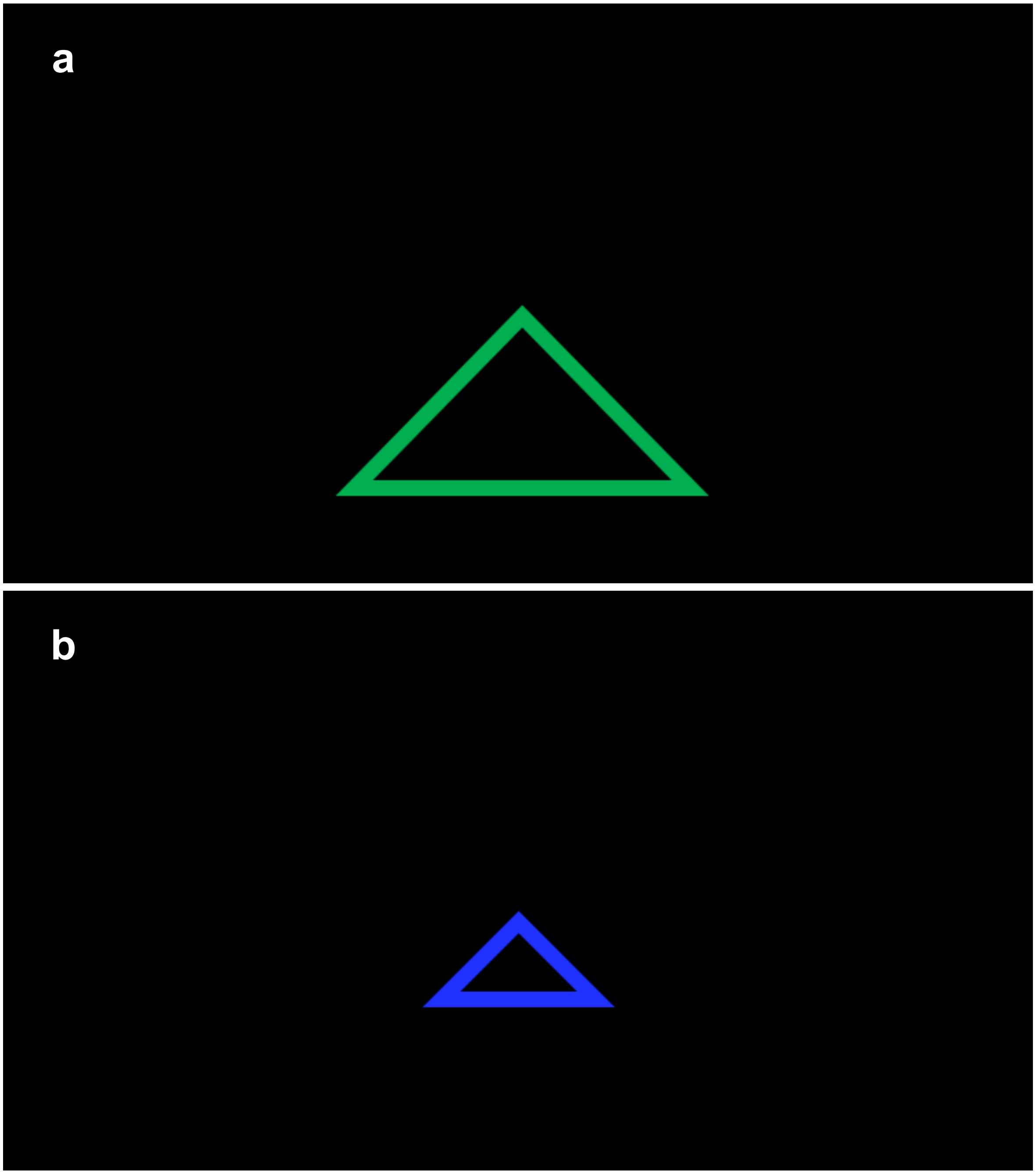

Supplementary Figure 25. Image projections of the triangular prism three-dimensional image. Images were projected into a 5 mM solution of **1** in CH_2Cl_2 with 1 ppm triethylamine. The Pro4500 UV projector was placed at focal length, which was 184 mm away from the center of the imaging chamber. The Miroir 720p projector was placed 114 mm away from the center of the imaging chamber. A 240–395 nm bandpass filter was mounted in front of the Pro4500 UV projector, and a 550 nm filter was mounted in front of the camera. The image was projected from the Pro4500 UV projector against a plane width of 64 pixels. (a) PowerPoint slide projected from the Miroir 720p projector. (b) PowerPoint slide projected from the Wintech Pro4500 UV projector.

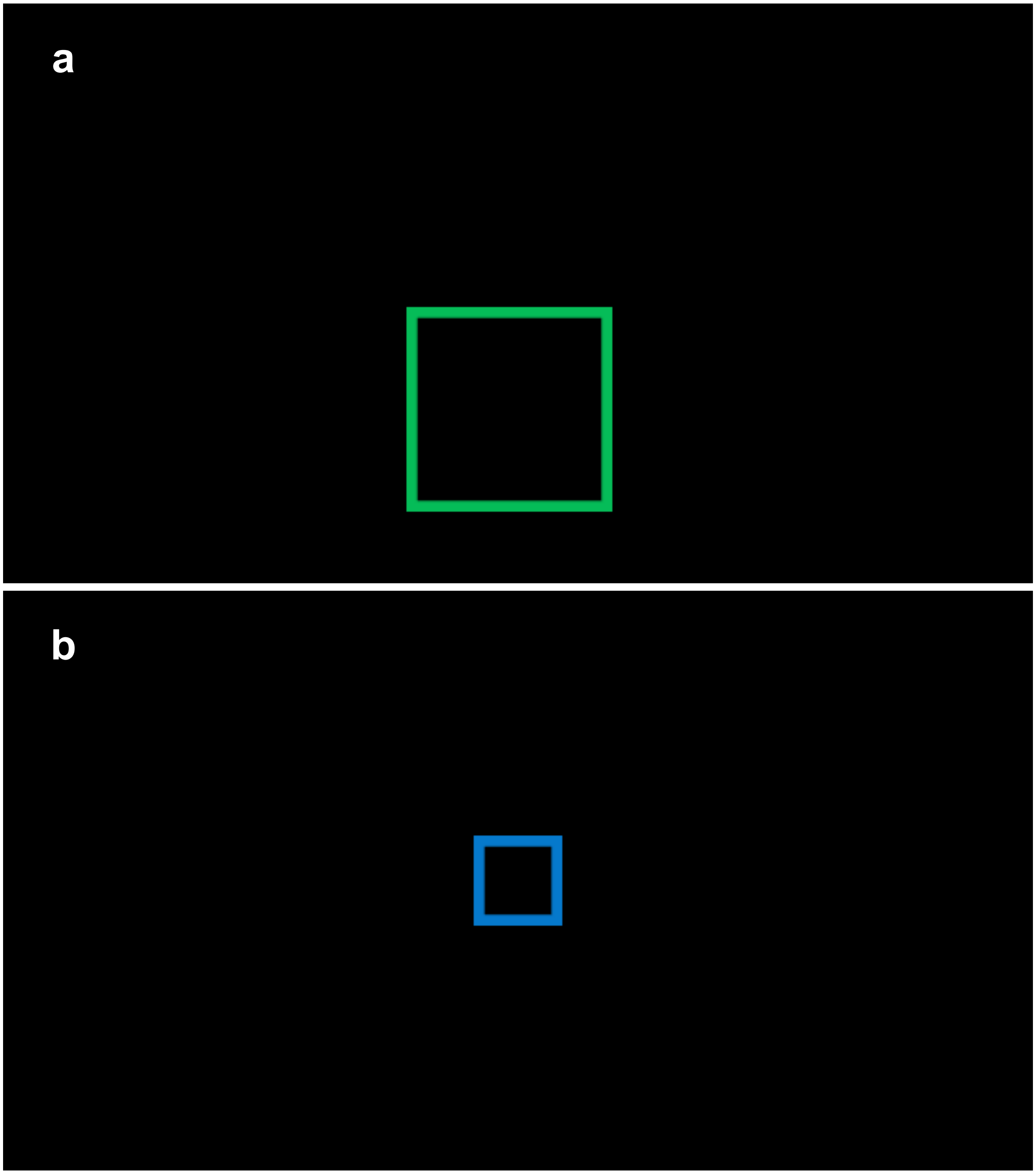

Supplementary Figure 26. Image projections of the three-dimensional image of the cube. Images were projected into a 5 mM solution of **1** in CH_2Cl_2 with 0.8 ppm triethylamine. The Pro4500 UV projector was placed at focal length, which was 184 mm away from the center of the imaging chamber. The Miroir 720p projector was placed 114 mm away from the center of the imaging chamber. A 240–395 nm bandpass filter was mounted in front of the Pro4500 UV projector, and a 550 nm filter was mounted in front of the camera. The image was projected from the Pro4500 UV projector against a plane width of 64 pixels. (a) PowerPoint slide projected from the Miroir 720p projector. (b) PowerPoint slide projected from the Wintech Pro4500 UV projector.

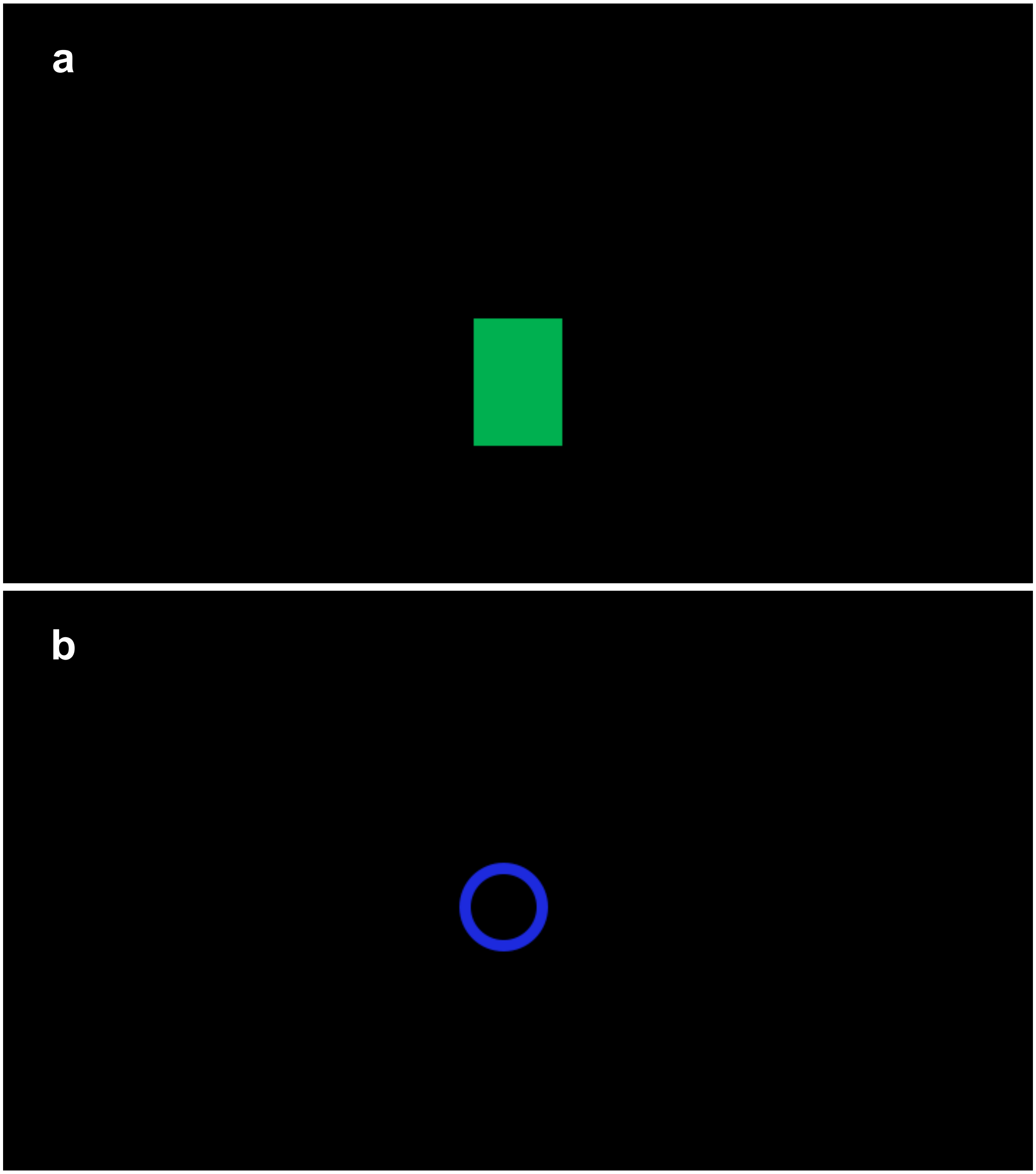

Supplementary Figure 27. Image projections of a three-dimensional image of a cylinder. Images were projected into a 5 mM solution of **1** in CH_2Cl_2 with 0.8 ppm triethylamine. The Pro4500 UV projector was placed at focal length, which was 184 mm away from the center of the imaging chamber. The Miroir 720p projector was placed 228 mm away from the center of the imaging chamber. A 240–395 nm bandpass filter was mounted in front of the Pro4500 UV projector, and a 550 nm filter was mounted in front of the camera. The image was projected from the UV projector against a plane width of 64 pixels. (a) PowerPoint slide projected from the Miroir 720p projector. (b) PowerPoint slide projected from the Wintech Pro4500 UV projector.

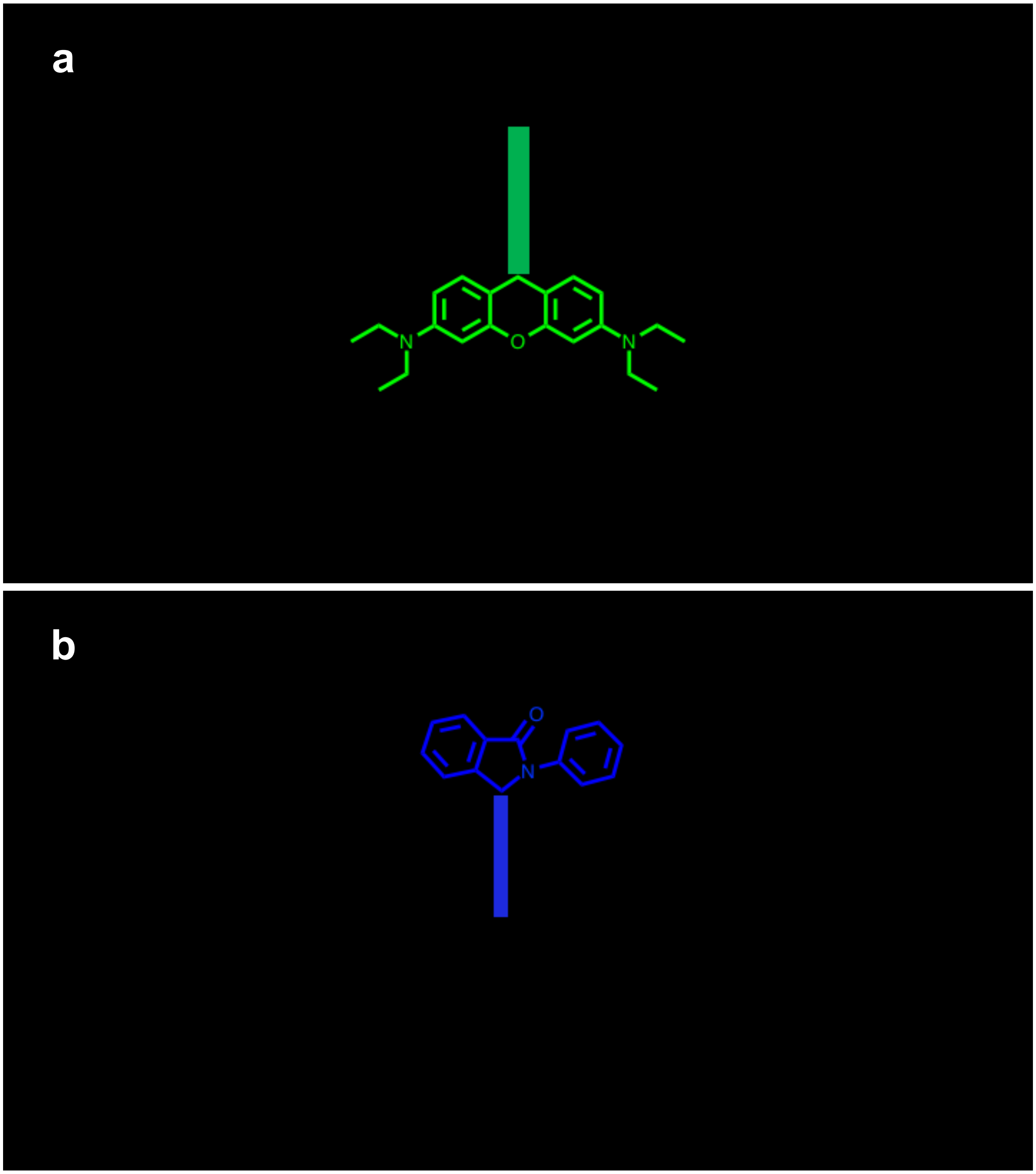

Supplementary Figure 28. Image projections of a three-dimensional image of the structure of **1**. Images were projected into a 5 mM solution of **1** in CH_2Cl_2 with 0.8 ppm triethylamine. The Pro4500 UV projector was placed at focal length, which was 184 mm away from the center of the imaging chamber. The Miroir 720p projector was placed 228 mm away from the center of the imaging chamber. A 240–395 nm bandpass filter was mounted in front of the Pro4500 UV projector, and a 550 nm filter was mounted in front of the camera. The image was projected from the Pro4500 UV projector against a plane width of 64 pixels. (a) PowerPoint slide projected from the Miroir 720p projector. (b) PowerPoint slide projected from the Wintech Pro4500 UV projector.

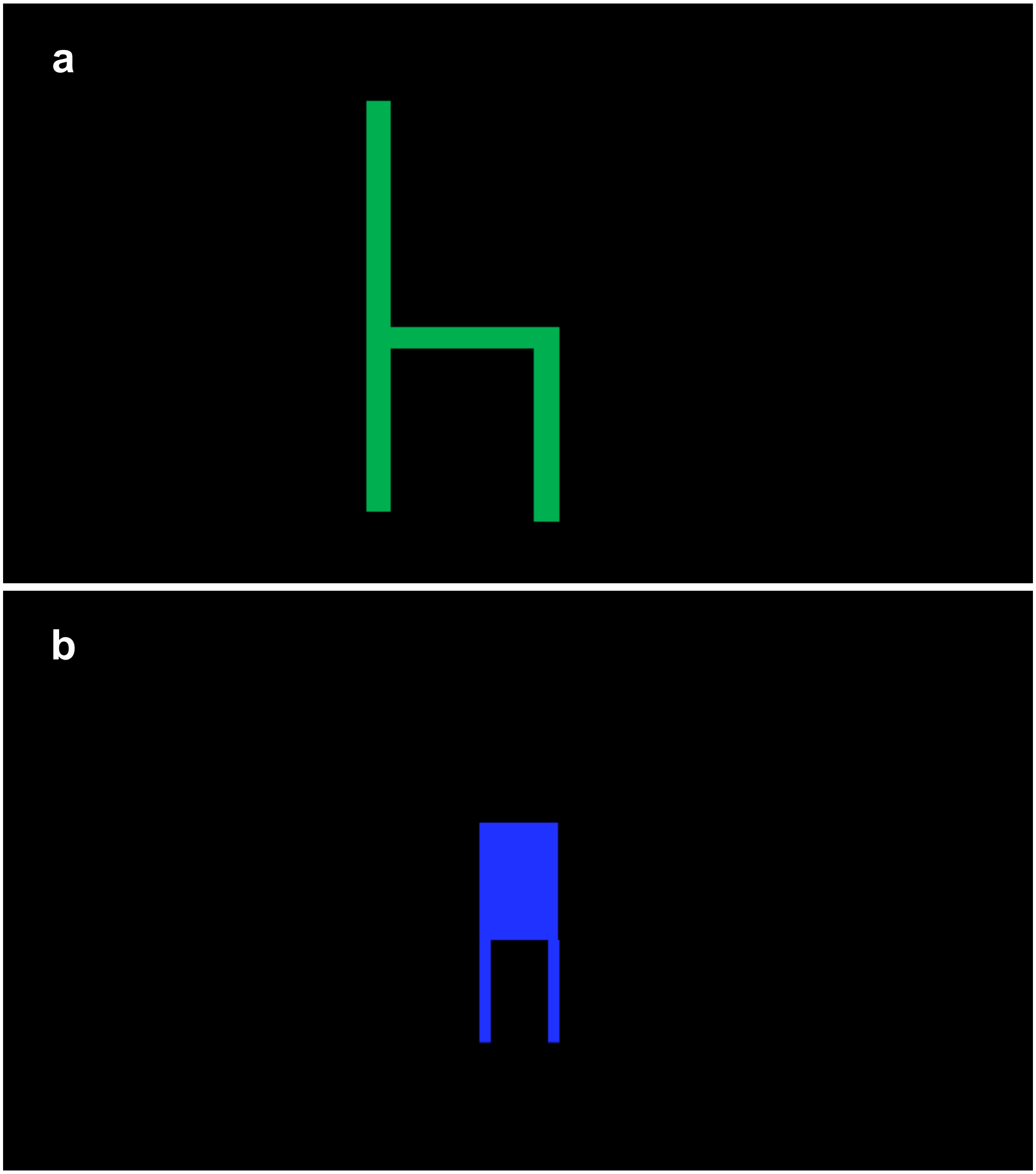

Supplementary Figure 29. Image projections of a three-dimensional image of a chair. Images were projected into a 5 mM solution of **1** in CH_2Cl_2 with 1 ppm triethylamine. The Pro4500 UV projector was placed at focal length, which was 184 mm away from the center of the imaging chamber. The Miroir 720p projector was placed 114 mm away from the center of the imaging chamber. A 240–395 nm bandpass filter was mounted in front of the UV projector, and a 550 nm filter was mounted in front of the camera. The image was projected from the Pro4500 UV projector against a plane width of 64 pixels. (a) PowerPoint slide projected from the Miroir 720p projector. (b) PowerPoint slide projected from the Wintech Pro4500 UV projector.

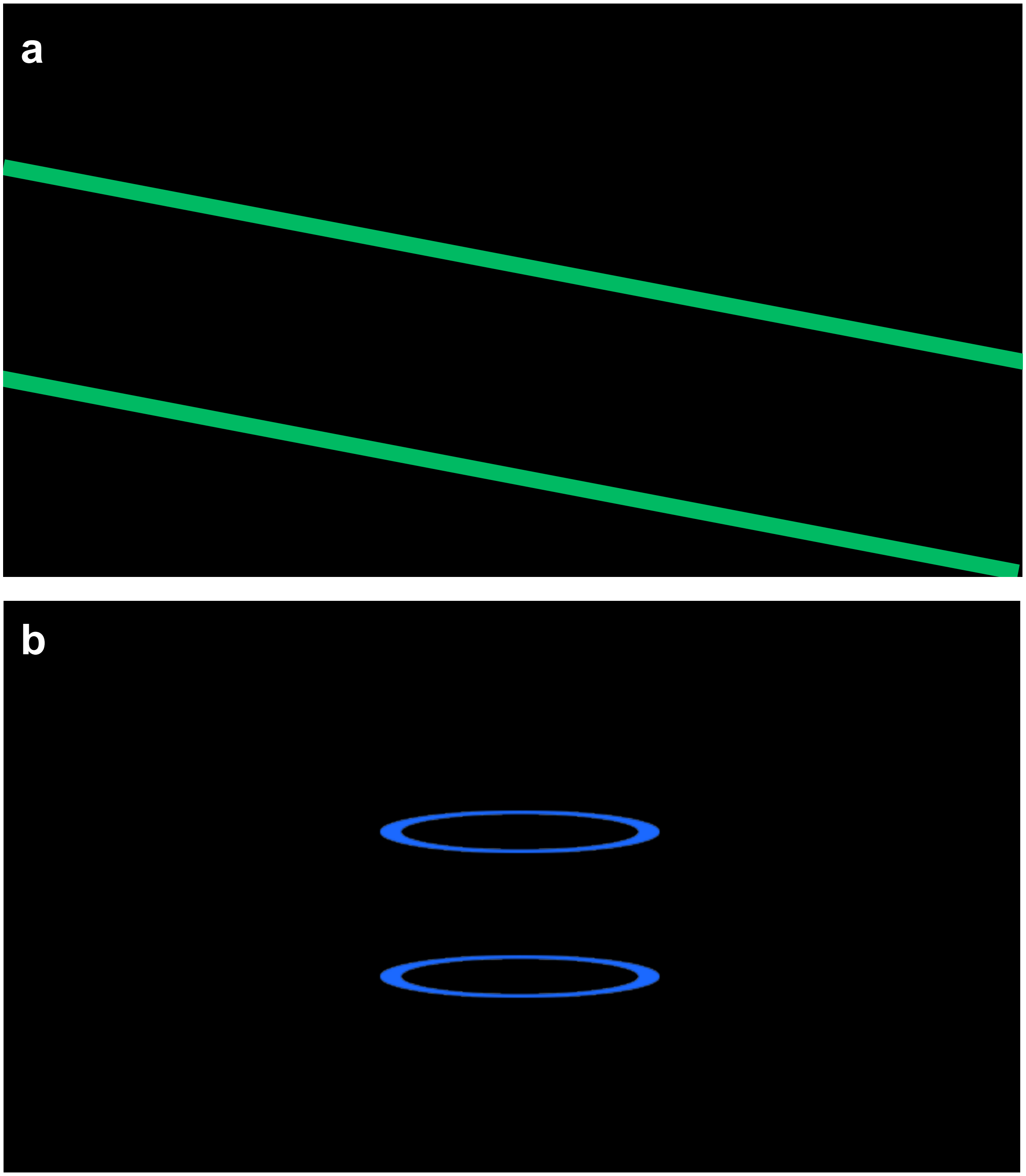

Supplementary Figure 30. Image projections of two stacked circles. Images were projected into a 5 mM solution of **1** in CH_2Cl_2 with 1 ppm triethylamine. The Pro4500 UV projector was placed at focal length, which was 184 mm away from the center of the imaging chamber. The Miroir 720p projector was placed 152 mm away from the center of the imaging chamber. A 240–395 nm bandpass filter was mounted in front of the Pro4500 UV projector, and a 550 nm filter was mounted in front of the camera. The image was projected from the Pro4500 UV projector against a plane width of 64 pixels. (a) PowerPoint slide projected from the Miroir 720p projector. (b) PowerPoint slide projected from the Wintech Pro4500 UV projector.

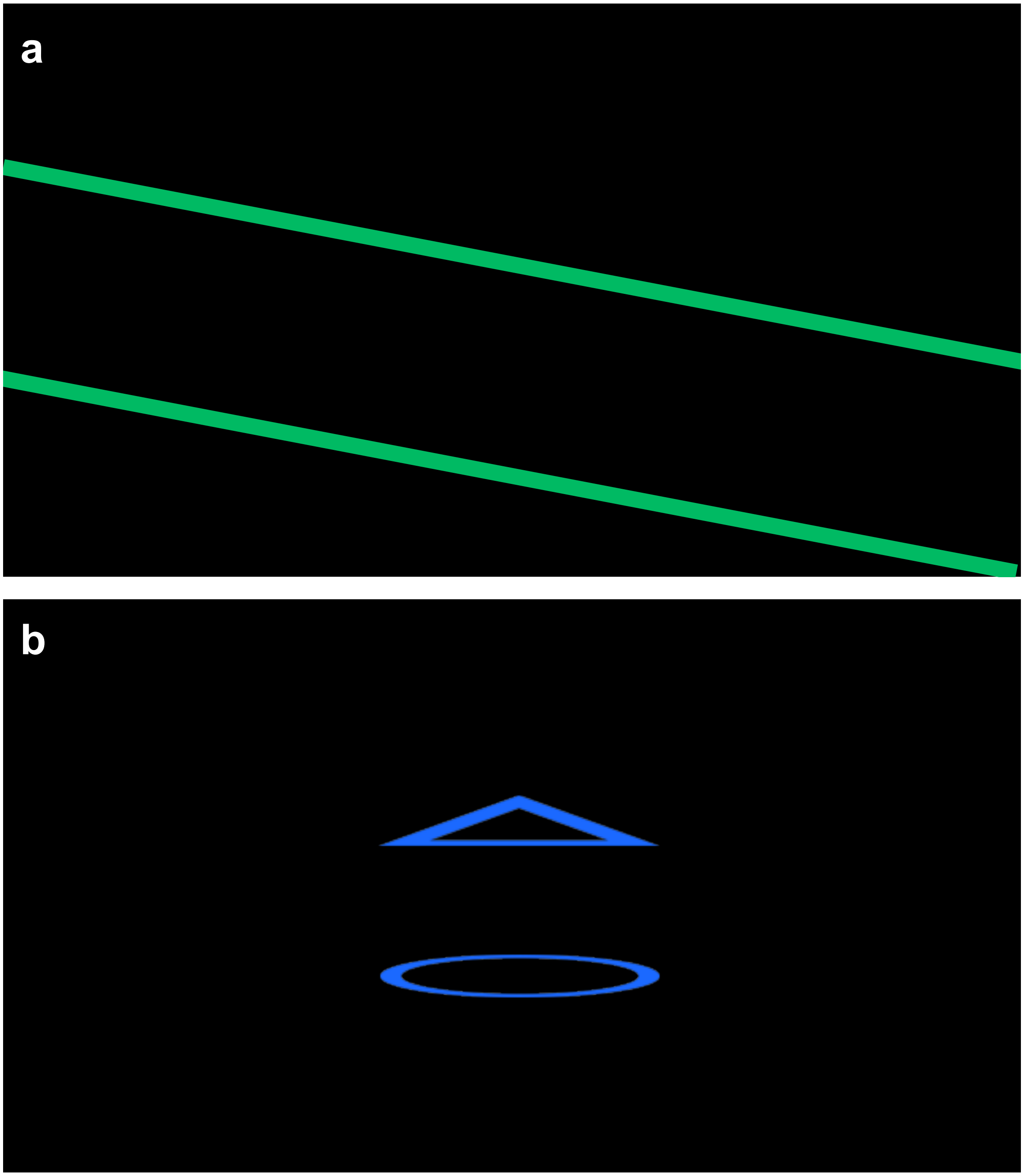

Supplementary Figure 31. Image projections of a triangle stacked above a circle. Images were projected into a 5 mM solution of **1** in CH_2Cl_2 with 1 ppm triethylamine. The Pro4500 UV projector was placed at focal length, which was 184 mm away from the center of the imaging chamber. The Miroir 720p projector was placed 152 mm away from the center of the imaging chamber. A 240–395 nm bandpass filter was mounted in front of the UV projector, and a 550 nm filter was mounted in front of the camera. The image was projected from the Pro4500 UV projector against a plane width of 64 pixels. (a) PowerPoint slide projected from the Miroir 720p projector. (b) PowerPoint slide projected from the Wintech Pro4500 UV projector.

Supplementary Table 1. Analysis of cost of supplies and materials based on actual expenditures.

Item	Expenditure
PRO4500 Wintech Optical Engine	$1,995.00
Miroir 720p HD Projector MP150	$419.95
ThorLabs Unmounted Bandpass Colored Glass Filter (240 - 395 nm)	$71.48
ThorLabs Unmounted Longpass Colored Glass Filter (550 nm)	$34.64
ThorLabs 1/2" Optical Post, SS, 8-32 Setscrew, 1/4"-20 Tap, L = 0.75", 5 Pack	$21.33
ThorLabs Right-Angle Clamp for 1/2" Posts, 3/16" Hex	$9.76
ThorLabs Aluminum Breadboard 12" x 18" x 1/2", 1/4"-20 Taps	$184.00
Custom Quartz Imaging Chamber	$1,290.00
Dichloromethane (2.5 L)	$40.60
Rhodamine B (500 gram)	$170.50
$POCl_3$ (250 gram)	$31.86
Aniline (100 gram)	$16.92
Triethylamine (2.5 L)	$89.31
**Total:**	$4,405

Supplementary Table 2. Comparison to other volumetric 3D displays.

Technology	Volume Type	Matrix	Light Source	Voxels	Volume	Resolution/Voxel size[a]
3D Light PAD[this work]	Static	Passive	DLP Projector	183,000[b]	125 cm^3	230 μm/0.68 mm^3 [b]
Solid-state upconversion[1]	Static	Passive	Laser (100 mW - 1 W)	N.D.[c]	3.4 cm^3	100 μm[d]/N.D.[c]
Laser scanning plasma[2]	Static	Passive	Laser (>1 W)[e]	N.D.[c]	N.D.[c]	100 μm[d]/ N. D.[c]
Ion-exchange waveguides[3]	Static	Active	Incandescent light bulb	2040	93 cm^3	3.8 mm/46 mm^3
Laser projected display[4]	Swept	Passive	Laser (200 - 300 mW)	800,000	25K cm^3	1.7 mm[d]/31 mm^3
Helical Screen DLP[5]	Swept	Passive	DLP Projector	157 million	62K cm^3	N.D.[f] / 0.39 mm^3
Actuality's Perspecta[6]	Swept	Passive	DLP Projector	100 million	21K cm^3	N.D.[f]/ 0.21 mm^3

[a] Voxel size determined by voxel size = volume / total voxels. [b] Estimated by dividing volume by voxel size. Voxel size was independently measured (Supplementary Fig. 15). [c] Not determined. [d] Determined as diameter of the focused laser spot. [e] Determined from values given in patent disclosure. [f] Not determined (resolution defined as number of voxels = 200 x 1024 x 768). [g] Not determined (resolution defined as number of voxels = 198 x 768 x 768).

Supplementary Methods

General methods and materials. All reagents were purchased from Sigma-Aldrich (St. Louis, MO), Alfa Aesar (Ward Hill, MA), or Acros Organics (Geel, Antwerp, Belgium) and were used without further purification. Reactions were performed in dried glassware under N_2 atmosphere. Column chromatography was performed on silica gel P60 (SiliCycle). Analytical thin layer chromatography (TLC) was used to monitor reactions and was performed on SiliCycle 60 F254 silica gel precoated sheets (0.25 mm thick). The light source used for spectroscopic experiments was a 150 W xenon lamp installed in a Hitachi F-7000 spectrophotometer (Hitachi, Tokyo, Japan). Absorbance measurements were taken on a Beckman Coulter DU 800 spectrophotometer

(Fullerton, CA) in quartz cuvettes (Starna, Atascadero, CA). Irradiation power was measured using a ThorLabs (Newton, NJ, USA) Energy Console Meter (PM100D) equipped with a Photodiode Power Sensor, 200–1100 nm (S120VC). ^{1}H NMR and ^{13}C NMR spectra were collected on a JEOL 500 MHz spectrometer in the Department of Chemistry at Southern Methodist University in $(CD_3)_2CO$ (Cambridge Isotope Laboratories, Cambridge, MA). Chemical shifts are reported in parts per million using the deuterated solvent proton signal as an internal reference. Coupling constants are reported in Hertz (Hz). Splitting patterns are indicated as follows: br, broad; s, singlet; d, doublet; t, triplet; q, quartet; m, multiplet; dd, doublet of doublets; dt, doublet of triplets.

Synthesis of 3',6'-bis(diethylamino)-2-phenylspiro[isoindoline-1,9'-xanthen]-3-one (1). Compound **1** was prepared by adaptation of a literature procedure.[7] Rhodamine B (1.0 equiv, 2.99 g, 6.24 mmol) and aniline (3.0 equiv, 1.7 mL, 19 mmol) were dissolved in dichloromethane (50 mL). The mixture was cooled to 0 °C, then $POCl_3$ (1.2 equiv, 0.70 mL, 7.5 mmol) was added dropwise. The reaction mixture was allowed to stir at 0 °C for 15 min and then heated to 45 °C for 20 h. The mixture was diluted with 50 mL dichloromethane and then washed with 1 M HCl (3 × 50 mL), 1 M NaOH (3 × 50 mL) and then washed with brine. The organic layer was collected, dried over Na_2SO_4, filtered, and concentrated under reduced pressure. The crude product was purified by silica gel chromatography with ethyl acetate:hexane (1:4) as the eluent, affording the product as a white solid (2.4 g, 75% yield). The compound can be additionally purified by recrystallization from 1:3 ethyl acetate:hexanes. ^{1}H NMR (500 MHz, $(CD_3)_2CO$) δ 7.87 (dd, J = 6.9, 1.2 Hz, 1H), 7.50–7.58 (m, 2H), 6.80–6.93 (m, 6H), 7.61 (d, J = 8.6 Hz, 2H), 6.40 (dd, J = 8.6 Hz, 2H), 6.27 (d, J = 2.3 Hz, 2H), 3.34 (q, J = 6.9 Hz, 8H), 1.11 (t, J = 6.9 Hz, 12H); ^{13}C NMR (125 MHz, $(CD_3)_2CO$) δ 166.91, 153.97, 152.99, 148.86, 137.70, 132.96, 130.70, 128.74, 128.24, 126.66, 126.11, 123.90, 122.84, 108.23, 106.56, 97.62, 66.82, 44.01, 12.00.

Evaluation of the irradiation time dependence of photoactivation. The effect of the duration of irradiation on absorbance was found by filling quartz cuvettes with 0.5 mL of a 5 mM solution of **1** in dichloromethane. The samples were illuminated with 315 nm light with the excitation slit opened to 20 nm and the emission slit set to 1 nm. The absorbance at 556 nm was measured after samples had been irradiated for 30, 60, 90, and 120 seconds (Supplementary Fig. 4). The light source used for these experiments was a 150 W xenon lamp in a Hitachi F-7000 spectrophotometer (Hitachi, Tokyo, Japan). Absorbance spectra were acquired using a Beckman Coulter DU 800 spectrophotometer.

Evaluation of the wavelength dependence of photoactivation. The wavelength dependence of the dye photoactivation was tested by filling quartz cuvettes with 0.5 mL of 5 mM solution of **1** in dichloromethane and illuminating the samples with light of varying wavelengths for one minute. The peak absorbance at 556 nm was measured after illumination with 245 nm, 265 nm, 285 nm, 305 nm, 325 nm, 365 nm, and 385 nm light. Absorbance spectra were acquired within 20 s after illumination (Supplementary Fig. 5). The light source used for these experiments was a 150 W xenon lamp in a Hitachi F-7000 spectrophotometer with the excitation slit opened to 20 nm and the

emission slit set to 1 nm. Peak absorbance was found using the Beckman Coulter DU 800 spectrophotometer.

Determining the rate of thermal fading. The absorbance at 556 nm of a 5 mM solution of **1** in dichloromethane was measured after irradiating with 315 nm light for varying times. The thermal fading rate was determined by fitting the decay to a single exponential similarly to Figure 1d of the manuscript (Supplementary Fig. 6).

Thermal fading kinetics in the presence of triethylamine. Solutions of 5 mM **1** in dichloromethane containing 1 ppm (7.2 μM), 5 ppm (36 μM), and 15 ppm (108 μM) triethylamine were prepared. For each test, the Pro4500 UV projector was placed at the focal length, or 184 mm from the middle of the imaging chamber, and the Miroir 720p projector was placed 127 mm away from the center of the imaging chamber. A UV square (25 x 25 pixels) was projected from the Pro4500 UV projector against a green plane (width of 49 pixels) for 10 seconds, and then the UV projector was switched to a blank slide for 10 seconds to allow the image to turn off. This cycle was repeated 3 times and recorded by a video using the stock smartphone camera app. The video was cropped for each cycle from the QuickTime application. Each cropped video was converted into an image sequence using MPEG Streamclip application. The image sequences were then uploaded into ImageJ (National Institutes of Health). The mean pixel intensity (MPI) of a region of interest was measured and graphed against time with each frame being a time interval of 33 ms. Observed rate constants were determined by fitting plots in Supplementary Fig. 7d and Supplementary Fig. 8d in Mathematica as described in the Methods section of the manuscript and the trends are plotted Supplementary Fig. 9.

Effect of triethylamine on contrast. Solutions of 5 mM **1** in dichloromethane containing 1 ppm (7.2 μM), 5 ppm (36 μM), and 15 ppm (108 μM) triethylamine were prepared. For each test, the Pro4500 UV projector was placed at the focal length, or 184 mm from the center of the imaging chamber, and the Miroir 720p projector was placed 101 mm away from the side of the imaging chamber. The projector GUI was set to video mode. LED driver control was set so that the red/green LED current was set to 0, the blue LED current was set to the arbitrary blue LED current value (*CV*) of 150. A UV square (25 x 25 pixels) was projected from the UV projector against a green plane (width of 49 pixels) for 10 seconds, and then the UV projector was switched to a blank slide for 10 seconds to allow the voxel to turn off. This cycle was repeated 3 times and recorded by a video using stock smartphone camera app. The video was cropped for each cycle from the QuickTime application. Each cropped video was converted into an image sequence using MPEG Streamclip application. The image sequences were then uploaded into ImageJ. The mean pixel intensity was found for the voxel (L_{max}) and for a background region that overlapped with the UV beam (L_{min}). Contrast for each solution was calculated using Supplementary Equation (1). The peak contrast of each set of images from the different concentrations of triethylamine was also found (Supplementary Fig. 10).

$$C = \frac{L_{max} - L_{min}}{L_{max} + L_{min}} \tag{1}$$

Effect of filters on contrast. Solutions of 5 mM **1** in dichloromethane containing 1 ppm triethylamine were prepared. The Pro4500 UV projector was placed at the focal length, which is 184 mm between lens and the center of the imaging chamber. The Miroir 720p projector was placed 101 mm away from the center of the imaging chamber. A square (25 x 25 pixels) was projected from the UV projector against a green plane (width of 49 pixels) for 5–10 seconds before images were taken. Pictures were taken with no filters, 515 nm long pass filter, 550 nm long pass filter, and 590 nm long pass filter mounted in front of the camera. Images were captured with and without a 240–395 nm bandpass filter mounted in front of the UV projector. Using the mean pixel intensity of the voxel and the mean pixel intensity from a region of interest centered on the off-voxel background of the UV beam as measured by ImageJ, contrast was found using Supplementary Equation (1). In Supplementary Fig. 14, contrast of the voxel for each filter tested has been reported without the 240–395 nm filter (the darker bar) and with the bandpass filter (the lighter bar).

Characterization of voxel size. A solution of 5 mM **1** in dichloromethane was prepared with 1 ppm triethylamine. For the tests in Supplementary Fig. 15, the Pro4500 UV projector was placed at the focal length (184 mm) away from the center of the imaging chamber and the Miroir 720p projector was placed 115 mm away from the center of the imaging chamber. For the tests in Supplementary Figs. 17–18, the Pro4500 UV projector was placed at the focal length (184 mm) away from the center of the imaging chamber and the Miroir 720p projector was placed 101 mm away from the center of the imaging chamber. The 240–395 nm bandpass filter was mounted in front of the UV projector, and the 550 nm filter was mounted in front of the camera. Square patterns of side lengths of 0 μm, 120 μm, 580 μm, 1.3 mm, 2.2 mm, 3.4 mm, 4.9 mm, 6.7 mm, 8.8 mm, 11 mm, 14 mm, 20 mm, and 27 mm were projected from the Pro4500 UV projector. For each set of UV squares, green plane widths of 0 μm, 160 μm, 300 μm, 670 μm, 1.1 mm, 1.8 mm, 2.6 mm, 3.5 mm, 4.6 mm, 5.8 mm, 7.2 mm, 11 mm, and 14 mm were projected from the Miroir 720p projector. Using the throw ratio reported by the projector manufacturers and the measured distance, the dimensions of the voxels were calculated. The voxel size has been arranged into a grid as seen from the image below (Supplementary Fig. 15).

Characterization of brightness and contrast. From the images collected from the voxel size experiment, the mean pixel intensity in a region of interest within the voxel was measured using ImageJ to determine the brightness (Supplementary Fig. 16a,b). The contrast was evaluated using Supplementary Equation (1), where L_{max} is the mean pixel intensity of the voxel (same as brightness) and L_{min} is the mean pixel intensity of a region of interest outside of the voxel region, but inside the path of the UV projection. In cases where a voxel could not be identified, the contrast was set to zero.

Evaluation of repeatability and fatigue resistance. Solutions of 5 mM **1** in dichloromethane containing 14.4 μM, 36 μM, or 108 μM triethylamine were added to the

quartz imaging chamber. A square was projected from the Pro4500 UV projector and a bar was projected from the Miroir 720p projector. The Powerpoint presentation projected from the UV projector was automated to change slides at a given interval and to play a "gunshot" sound as each slide was changed. This "gunshot" sound was used as an audio trigger for a smartphone photography application. Two to four photographs were taken for each "on" and "off" image and 75–250 cycles were analyzed by measuring the mean pixel intensity of the "on" and "off" states (Fig. 5c, Supplementary Fig. 19). The fatigue resistance over sequential days was determined by continuously illuminating a UV square from the Pro4500 projector and a green bar from the Miroir 720p projector into solution containing 5 mM **1** in dichloromethane containing 7.2 μM triethylamine. The illumination was continued for 1 hour and the brightness and contrast were determined from the mean pixel intensity (MPI) of the image and background. This was repeated using the same solution on 8 days over an 11-day period (Supplementary Fig. 20).

Details of image projections. For each of the image projections described in Supplementary Figs 21–31, the green images (a) are the slides projected using PowerPoint from the green Miroir 720p projector, and the blue images (b) are the slides projected using PowerPoint from the Pro 4500 UV projector. For photographs, the image was projected from the Pro4500 UV projector without altering the color of the photograph.

"Moving cube" animation conditions. The animation was projected in a 5 mM solution of *N*-phenyl spirolactam rhodamine B **1** in dichloromethane containing varying amounts of triethylamine (1 ppm, 5 ppm, and 10 ppm). The Pro4500 UV projector was placed at the focal length (184 mm) away from the voxel and the Miroir 720p projector was placed 179 mm away from the center of the imaging chamber. The 240–395 nm bandpass filter was mounted in front of the UV projector, and the 550 nm filter was mounted in front of the camera. Projector GUI set to video mode. LED driver control was set so that the red/green LED current was set to 0, the blue LED current was set to the arbitrary blue LED current value (*CV*) of 150.

"Running horse" animation conditions. The animation was projected in a 5 mM solution of *N*-phenyl spirolactam rhodamine B **1** containing 0.8 ppm triethylamine. The images were projected from the Miroir 720p projector against a UV plane that was 16 pixels in width. The Pro4500 UV projector was placed at the focal length (184 mm) away from the voxel and the Miroir 720p projector was placed 228 mm away from the center of the imaging chamber. The 240–395 nm bandpass filter was mounted in front of the UV projector, and the 550 nm filter was mounted in front of the camera. The projector GUI was set to video mode. The LED driver control was set so that the red/green LED current was set to 0, the blue LED current was set to the arbitrary blue LED current value (*CV*) of 150.

Supplementary References

1. Downing, E., Hesselink, L., Ralston, J. & Macfarlane, R. A three-color, solid-state, three-dimensional display. *Science* **273**, 1185–1189 (1996).
2. Momiuchi, M. & Kimura, H. (Burton Inc.) Device for forming visible image in air. U.S. Patent 7,533,995, filed August 12, 2004, and issued May 19, 2009.
3. Penciu, C. & MacFarlane, D. L. Fabrication and characterization of a volumetric three-dimensional display using ion-exchange integrated waveguides. *Opt. Eng.* **39**, 565–571 (2000).
4. Lasher, M., Stolan, P., Dahlke, W., Acantilado, N. & McDonald, M. Laser Projected 3-D Volumetric Displays. *Proc. SPIE* **2650**, 285–295 (1996).
5. Geng, J. A volumetric 3D display based on a DLP projection engine. *Displays* **34**, 39–48 (2013).
6. Favalora, G. E., Napoli, J., Hall, D. M., Dorval, R. K., Giovinco, M. G., Richmond, M. J. & Chun, W. S. 100 Million-voxel volumetric display. *Proc SPIE* **4712**, 300–312 (2002).
7. Best, Q. A., Liu, C., van Hoveln, P. D., McCarroll, M. E. & Scott, C. N. Anilinomethylrhodamines: pH sensitive probes with tunable photophysical properties by substituent effect. *J. Org. Chem.* **78**, 10134–10143 (2013).
